# Sustainable Valorization of Spent Coffee Grounds: A Green Chemistry Approach to Soil Amendment and Environmental Monitoring

**DOI:** 10.1021/acssusresmgt.5c00083

**Published:** 2025-09-16

**Authors:** Ashvinder Kumar, Manju K. Thakur, Phil Hart, Vijay K. Thakur

**Affiliations:** a Biorefining and Advanced Materials Research Center, SRUC, Kings Buildings, West Mains Road, Edinburgh EH9 3JG, UK; b Department of Chemistry, RNT Government College, Sarkaghat, Mandi, HP 175024, India; c The School of Water, Energy and Environment (SWEE), Cranfield University, Cranfield Bedfordshire MK43 0AL, UK; d Renewable and Sustainable Energy Research Centre, 559361 Technology Innovation Institute, P.O. Box 9639, Abu Dhabi 9639, United Arab Emirates

**Keywords:** spent coffee grounds, fulvic acid, biochar, compost, nutrient, soil fertility

## Abstract

Nowadays, soil is deteriorating at an alarming rate, endangering both land fertility and productivity and thus the world’s food supply. Using bulk available spent coffee ground (SCG) solid wastes to enrich and amend the deteriorating soil might be revolutionary because it would assist with its correct disposal and lessen the problems related to environmental contamination and human health. The blend of traditional practices and modern technologies can manage SCG’s waste economically, efficiently, and sustainably. The current review article focuses on the potential uses of wasted coffee grounds to improve soil fertility, water-holding capacity, residue management, seed germination, crop growth, and yields. The ability of SCG to amend soil depends upon the nature of SCG (fresh, compost, vermicompost, biochar, etc.), mode of application (extract, mixing, and top dressing), and application rate. The traditional practice of composting using microbes and earthworms to convert phytotoxic SCG into non-phytotoxic compost to enhance crop productivity and soil fertility is quite impressive and has been applied extensively. However, other modern technologies, like SCG-derived biochar, hydrochar, alkaline-treated SCG, SCG-derived nano fulvic-like acid fertilizers, and NPK-organic fertilizers, could be an excellent choice to replace the existing ones. This paper details the recent advancements and effects of various fertilizers on the physicochemical characteristics of soil, compost nutrient composition, plant growth, nutrient uptake by plants, and soil’s ability to store water.

## Graphical abstract

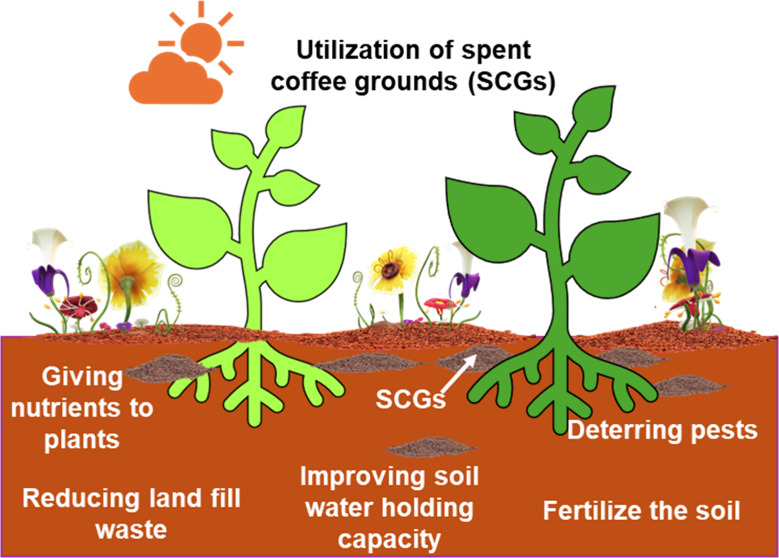

## 1 Introduction

Over the last few decades, farmers have been using inorganic fertilizers rich in high nutrients to enhance soil fertility to fulfill the rising food demand of the growing population. However, persistent use of inorganic fertilizers has resulted in several adverse impacts on the environment, including soil erosion, eutrophication, soil acidification, soil deterioration, loss of biodiversity, and greenhouse gas emissions.^1–3^ Its overutilization generally destroys soil decomposers, i.e., fungi, earthworms, bacteria, and other living organisms in the soil, which in turn reduces the colonization of plant roots and thereby prevents rhizobia from engaging in symbiotic N-fixation because of high N fertilization.^4–6^ Inorganic fertilizers impact soil health and lead to poor physical environments and human health. To reduce the negative environmental impacts of these chemical fertilizers, numerous sustainable management practices have been developed and utilized, such as fully green organic fertilizers and mixture of organic and inorganic fertilizers.^1,3^ Among these, organic amendments like plant residue, manure, organic waste-derived compost, and vermicompost have attracted worldwide attention because of their remarkable ability to enhance soil fertility, provide high crop yields, and mitigate greenhouse gas emissions.^7–9^

According to research by Haque et al.,^10^ adding manure to the soil can decrease the need for inorganic fertilizers, leading to a significant boost in rice yield. Similarly, Zoghlami et al.^11^ demonstrated how adding sludges to the soil of semi-arid regions can enhance soil’s structural stability and fertility, which not only increases soil organic matter contents but also enhances macro- and micronutrients.

Coffee is the second most traded product (produced in 80 countries) and one of the most popular beverages in the world.^12^ Over the last 30 years, its production has risen by more than 60% (ICO, 2019), and annual cross-border exports have grown from 8.4 to 35.6 billion USD from 1991 to 2019, respectively. Spent coffee grounds (SCGs), a final product after the coffee brewing process, despite having numerous desirable chemical components, are usually considered waste and are generally thrown away or composted. The chemical composition of most of the used coffee grounds is quite similar; however, coffee grounds prepared to make instant coffee possess a lower percentage composition of chemical compounds because of the more extensive extraction process.^13^

It has been reported that, on average, 650 kg of SCG is generated from 1 tonne of green coffee,^14^ and approximately over 15 million tonnes of SCGs are created annually.^15^ Most of the SCGs are disposed of in landfills, which triggers the risk of spontaneous combustion and also produces harmful air-polluting gases like CO_2_ and CH_4_ in excessive amounts.^16,17^ Thus, it is of utmost necessity to recycle/reuse this abundantly available and low-cost solid waste by employing different valorization techniques (Figure 1). The Food and Agriculture Organization of the United Nations (FAO) has also mentioned that recycling of these organic wastes is critical to achieve a green, sustainable, and circular bioeconomy (FAO, 2020). Thus, there is an urgent need for the development of a waste management plan consistent with existing national regulations to meet the life cycle approach to sustainability.^18^

**Figure 1:**
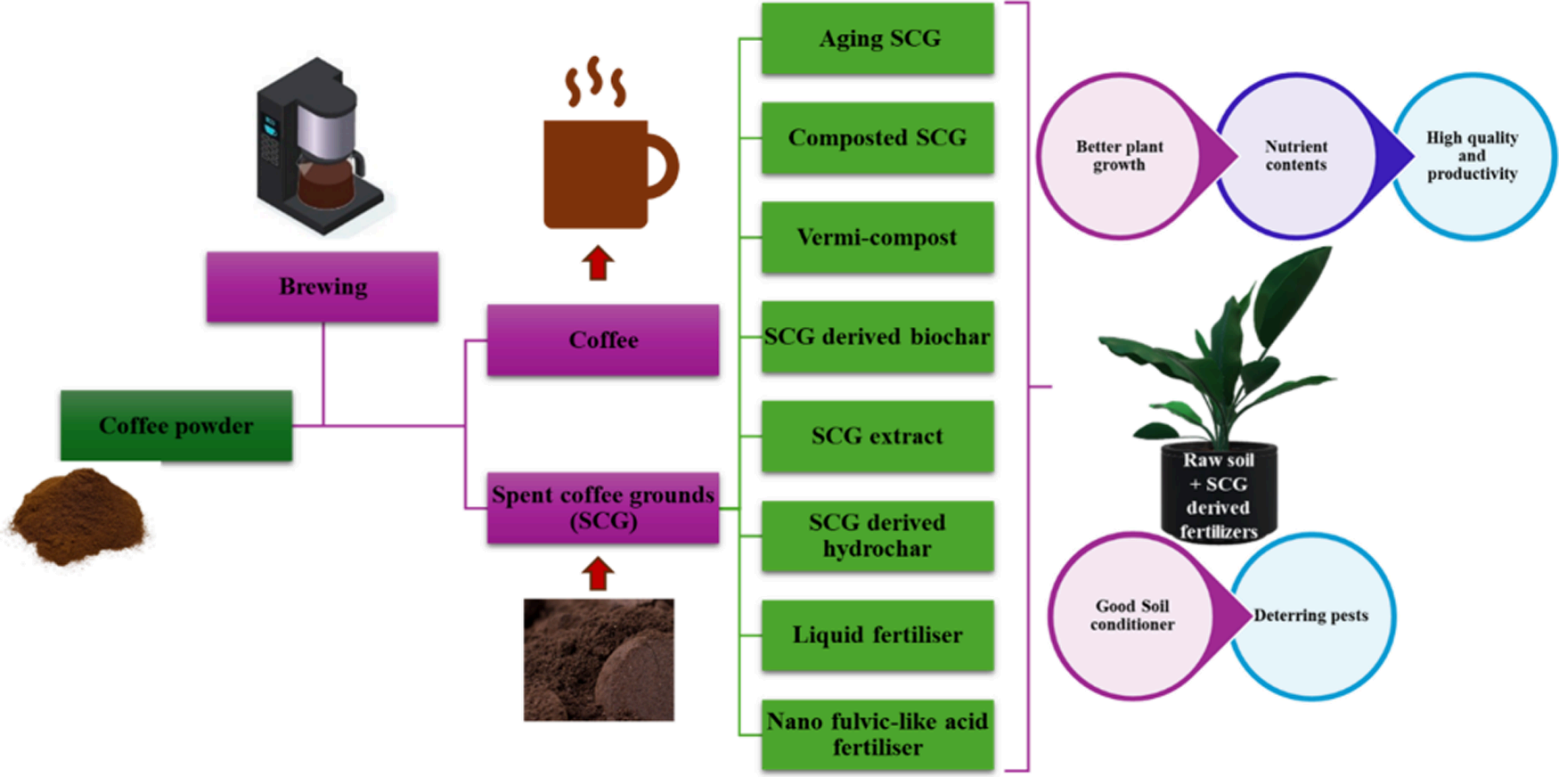
Depicting different types of SCG-derived fertilizers and their effect on plant growth.

When we prepare coffee, it hardly impacts the properties of SCG’s constituents like cellulose, minerals, polyphenols, and lipids.^18^ Recently, numerous efforts have been made to use waste coffee to produce biofuels and hydrogels for multiple applications, such as compost. A UK-based company named “Bio-bean” is recycling SCGs into biomass pellets and advanced biofuels^19^ and has been recently announced as the B Corp Best in the World Environment.^20^ One more company named “EcoBean” has just started up in Poland, which is deriving chemicals like coffee oil, antioxidants, lignin, and protein additives from SCGs.^21^ It has been reported that around 9000 tonnes of SCGs are wasted in Europe every day, and approximately 3.3 million tonnes of SCGs are fueling Europe’s CO_2_ problem each year. eNvar company, in addition to biofuel extraction, is also developing bio-fertilizer pellets from SCGs for the horticultural, agricultural, and viticultural markets.^22^ A Dubai-based start-up “Bean & Beyond”, started in 2022, partnered with RAW Coffee Company to produce gourmet oyster mushrooms from SCGs in the UAE.^23^ In addition, some more famous companies like UpCircle, Rens, Kaffe Bueno, and Gro Cycle are also using SCGs to prepare natural skin care products, lightweight shoes, and different caffe products (kaffee oil: for anti-aging, wound healing, moisturizing, and hair care; kafflour: protein-rich gluten-free flour for bakery; and kaffibre: used for exfoliating shrubs and face-masks in the beauty industry) and to grow oyster mushrooms, respectively.^24^ Chung et al.^25^ utilized SCG as an additive in mill clay, activated using a mixture of NaOH and Na_2_SiO_3_, to produce eco-friendly unfired bricks. In Italy, approximately 4.5 million tons of eco-bricks are produced annually for residential construction (masonry roofing, horizontal structuring, partitioning of floors), which shares a market value of about 350 million euros.^26^ Forcina et al.^26^ analyzed and concluded that SCG replaces 3% of the total weight of clay, and thus, 1 ton of SCG will be able to produce around 33.3 tons of eco-bricks. The bricks loaded with 1–2.5 wt % of SCGs was found to possess a minimum desirable compressive strength of 8.5 MPa, which ensures better performance of the bricks; however, upon a further increase in its loading, a compromise in the functionality of the bricks needs to be made.

Furthermore, SCGs, because of the presence of a large amount of nutrients and low risk of contamination (heavy metals are absent), have also been used for amendments of soil and/or dressing of the top layer of soil to control weeds and soil quality^27–29^ (Figure 2). When used to feed the soil microbes, microbial glues are released, which further promote soil structure and enhance drainage.^30^ Upon utilizing an excessive amount of SCGs without composting, multiple effects on the soil texture can be noticed, and caffeine residues left over after immobilization can suppress the germination of seeds and the growth of plants. In a compost pile or bin, SCG is generally composted by layering three parts of leaves from one part of fresh grass to one part of SCGs, limiting the SCG volume below 20% by volume. However, it can be toxic to plants. SCGs have also been composted with animal manure and other organic wastes to enhance plant growth without intoxicating the soil.^31,32^ A few review articles have already been published on the valorization of SCGs.^33–35^ However, in the present review article, the role of SCGs as composting and soil amending agents in agriculture fields has been thoroughly reviewed.

**Figure 2:**
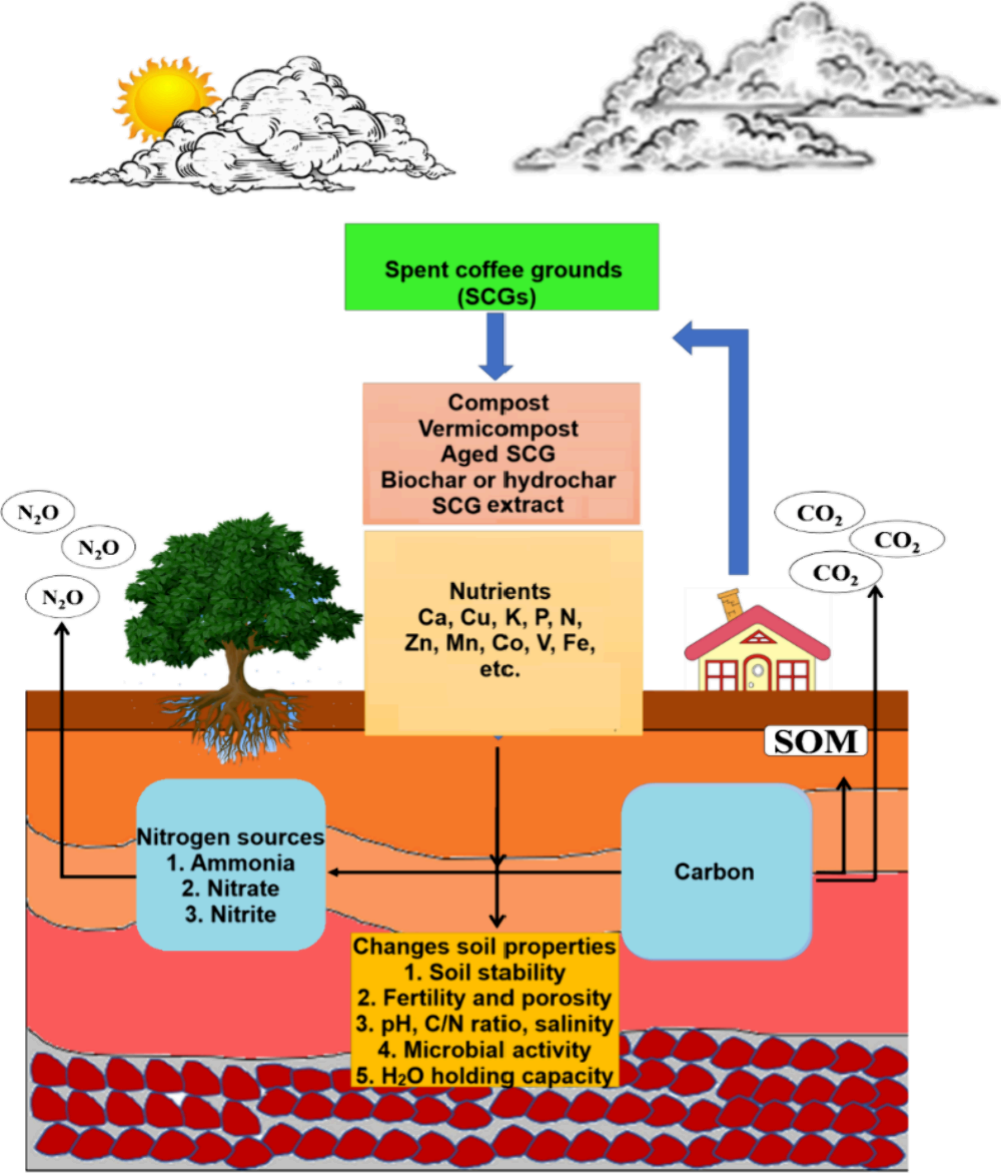
Systematic view of various processes during soil amendments of soil using SCG.^36^ Figure redrawn from ref 36 under Creative Commons Attribution International Licence (CC BY 4.0).

## 2 Composition

The composition of SCG varies with the type of coffee, brewing method, and growing conditions.^37^ The comparative view of the chemical composition of SCGs studied by different researchers is depicted in Table S1. From the table, it can be analyzed that most SCGs possess a similar chemical composition.^38–43^ The most significant polysaccharide component of SCG is hemicellulose, followed by lignin and cellulose. The hemicellulose (main constituents are galactose, arabinose, and mannose) and cellulose (glucose being the main constituent) collectively comprise 50% of the total dry mass of the SCG.^39^ Lignin is a brown-colored complex high molecular weight organic polymer that provides rigidity and remains as part of the SCG after brewing. However, the brown pigmentation of SCG grounds is mainly due to melanoidins rather than the lignin.^44^ Melanoidins are typically brown-colored complex polymeric compounds that are generated through Maillard reaction, a reaction between reducing sugars and amino acids during brewing and roasting. These melanoidins differ structurally from lignin and play a major role in the dark color of the SCGs. Protein is another major constituent of SCG, accounting for approximately 20% of dry SCG.^39^ In addition, it also contains a small amount of lipids, ash, caffeine, phenolics, and tannins (1.2–1.5%).^37,40,45–47^ Low et al.^45^ and Vandeponseele et al.^46^ reported a recovery of 21.02% tannic and 0.0046% caffeine, respectively. The presence of tannins, caffeine (0.02–0.08%), and phenolic compounds makes SCG unsuitable for direct application as soil amendments, as it impacts physiological responses like water uptake behavior, cellular expansion, mobility of nutrients, and soil–plant relations for respiration and consequently inhibits plant growth.^48^ Cervera-Mata et al.^49^ found an increase in vega and red soil respiration rates from 0.270 to 3.309 mg CO_2_/g day: 0.248 to 3.383 mg CO_2_/g day, respectively, and total phenolic compounds from 0.001 to 4.122 mg GAE/g, 0.353 to 6.309 mg GAE/g after the addition of 10% SCG. No doubt, the higher phenolic compounds in SCG may be toxic to plants and soil microbes’ health, but they can also be used as natural herbicides and pesticides.^50^ Several researchers have degraded these compounds by employing composting, vermico-composting, and chemical oxidation techniques. The details of this have been discussed in subsequent applications. Hachicha et al.,^51^ when co-composing the SCG and subsequently inoculating it with white-rot fungus *Trametes versicolor*, reported a 72% decrement (decline from 0.6 ± 0.1 to 0.2 ± 0.05%) in polyphenol contents and a 65% reduction in C/N ratio (decreased from 42 ± 5 to 14 ± 2) and an enhancement in humic acid concentration from 7 ± 1 to 28 ± 1% compared to control samples. Their results confirmed why composting or vermicomposting is necessary for SCG, prior to its utility in the agriculture field.

Table S1 also summarizes the elemental composition of SCG and SCG-derived fertilizers. Numerous mineral elements like K, Ca, Mg, S, P, Fe, Mn, B, Cu, Zn, and Ni were noted to be present in the SCG-derived ashes or growing media and mineralization filtrates.^31,31,39–41,49,39–41,52–58^ Most of these minerals present in SCG or SCG-derived compost are essential for human health since they regulate various physiological and metabolic functions of the human body, such as growth, enzymatic activities, and electrolyte balance.^59^ K is the most abundant mineral element, followed by Ca, Mg, and P. In addition to controlling enzymatic activities, these minerals also play a vital role in processes such as digestion, respiration, and circulation. Thus, the presence of these micronutrients in SCG should be capitalized to produce nutrient-enriched meals. From the table, it can also be concluded that heavy metal contents lie far below the threshold of potentially toxic levels established by regulations (Spanish Royal Decree 506/2013), further supporting their role in advanced agriculture.^55^

## 3 Impacts of Spent Coffee Ground-Derived Organic Fertilizers on Seed Germination, Seedling, and Plant Growth

Various processes for the direct application of SCG in agriculture are typically not advised since the presence of ecotoxic caffeine, polyphenols, and tannins in SCG inhibits seed germination and crop growth^28,49–51,60,61,49–51,61–72^ (Table S2.1). Several researchers reported negative impacts of fresh SCG on the growth and development of plants. When mixed with topsoil, a decrease in growth of various plants was noted, namely, lettuce, broccoli, cress, sunflower, leek, viola, and radish.^49,50,60,61,73,74^ Benefiting from their growth-inhibiting effect, numerous researchers proposed utilization of fresh SCG to control the weeds in field crops.^28,63,73^ Additionally, to lessen the negative impacts on the crop while still repressing weeds, fresh SCG has been applied as a mulch after crop seedling.^63^ Furthermore, the inhibitory effect of SCG was reported to diminish after 1 year in a field trial by Yamane et al.^73^ and after 3 to 4 months in a pot experiment by Nababi et al.^75^ Therefore, detoxification of SCG is necessary before its use as a soil amendment (Tables S2.2–S2.5). In this regard, composting/vermicomposting techniques have been reported to be quite effective, causing humification and detoxification of SCG, leading to various seed germination/plant growth-promoting end products.^62,73^ Dimitrijević et al.^76^ evaluated the impact of a number of composting days (ranging from 0 to 91 days) on the ability of SCG-derived compost towards germination of *Fagopyrum escultentum*. All samples (compost derived from varying amounts of SCG; 1–10 wt % with respect to medicinal plant waste) exhibited phytotoxic activity [germination index (GI) < 65] during the first 36 days of composting; however, compost was found to be acceptable after 42 days as it showed a GI higher than 70%. After the 91st day, compost was found to be non-phytotoxic, and the highest GI was noted with compost samples PBC5% [mixture treated with 2% v/w with bacteria; GI: 127.2 ± 4.2%], followed by PBC10% (114.7 ± 4.3%). Phytotoxicity of compost during the initial stages of the composting process might be because of the presence of ethylene oxide, NH_3_, and organic acids, as these gaseous substances severely affect seed germination and growth of the plant roots. A couple of researchers have also reported the release of four phytotoxic gaseous substances methane, carbon dioxide, nitrous oxide, and ammonia during the decomposition of feedstock.^77,78^ Generally, if the substrate possesses GIs less than 25, then it is considered to be phytotoxic; between 26 and 65, it confirms its mild or moderate phytotoxicity; in the range of 66 to 100, it means the substrate is phytotoxic; and a value above 100 confirms the non-phytostimulant and phytonutrient status of the substrate.^79^ Numerous researchers have carried out composting of SCG to lessen the phytotoxic impacts and to enhance the GI index/plant growth of various crops, the details of which are summarized in Table S2.4.^53,55,75,80–89^

Some researchers employed aged SCG to amend the soil and reported an increase in plant growth and a reduction in slug herbivory.^62,65^ Kekelis et al.^90^ evaluated the effect of different concentrations (1, 2, and 4% w/v) of SCG on soil free-living nematode communities in two different soils, namely, clay and sandy loam, for 3 and 6 months. No toxic impacts of SCG incorporation on the soil’s free-living nematodes have been noted; in fact, it was found to favor the nematode populations with the highest growth in sandy loam soil type. In all soil samples treated with different SCG contents, Panagrolaimus was dominant, indicating high soil enrichment. However, after 6 months of application, SCG was reported to decompose in 1 and 2 % treatment, confirmed by studying the nematode community structure indices, indicating the stage of nutrient depletion. Thus, when SCG is less than 4%, a second application is generally preferred within 6 months to avoid soil degradation. Furthermore, the nematicide potential of SCG against plant parasites has also been confirmed. Hydrochar^91^ and biochar^29,92–94^ derived from SCG were also used as eco-friendly alternatives for soil amendment, which enhanced soil fertility (microorganisms showed promising response) and/or abiotic parameters; however, their utility can increase the risk of increased greenhouse gas emissions and disease and pest outbreaks (Table S2.3).

Recently, Alghamdi et al.^93^ utilized the spent Arabica (SAW) and Columbian coffee (SCW) waste-derived biochar (SAWB and SCWB) to amend the sandy soil. During the greenhouse experiment, they found that a 5% application of SAWB and SCWB amendments enhanced the maize root biomass from 0.51 g (control treatment) to 2.12 and 2.38 g, shoot biomass from 7.37 to 9.70 and 9.93 g, and increased plant height from 15.71 to 30.94 and 33.23 cm, respectively. Cervera-Mata et al.^94^ used six different treated SCGs, i.e., composted, vermicompost (vermicomposting of SCG using *Eisenia foetida*, red earthworms), SCG-derived biochar, and ethanol-treated, water-treated, and hydrolyzed SCG in the proportion of 7.5% to vega soil to enhance the productivity of lettuce plant. They found the highest wet mass of lettuces grown in vermicocompost, followed by biochar. However, a decrease in the amount of Zn, Cu, and Fe in lettuce plants was noted when grown in vermicompost or biochar-mixed soil because of microbial/thermal degradation of natural chelating compounds. Furthermore, vermicompost and compost significantly enhanced the EC25 of soil because of higher salinity (greater than 20 Ds/m), giving more salinity than raw SCG. Darwis and Lisdiana^95^ reported improvement in seedling height, number of leaves and branches, dry weight, stem diameter, and leaf area of *Capsicum frutescens* L. with increased SCG-derived vermicompost. A dosage of 25 g/5 kg led to maximum growth of *Capsicum frutescens* L. breeding. When extract derived from SCG-based compost^50,51,53,76,96^ torrefied SCG^82^ and vermicompost^76^ were employed for seed germination, a notable enhancement in GI and seedling was noted (Table S2.5).

Zhang and Sun^83^ used a combination of cow dung (CD) and SCGs to enhance the degradation and humification of green waste (GW) during composting. When GW was composted with 20% CD and 45% SCGs, the highest quality and most mature compost product was produced in only 21 days. The combination of CD and SCG enhanced the thermophilic phase and enzyme activities, leading to the generation of low-molecular-weight compounds, HA (humic acid) concentration, decreased NH_4_^+^–N/NO_3_^–^–N ratio, N loss, and lignocellulosic substances and generated non-phytotoxic compost products with enhanced mechanical toughness, particle size distribution, and nutrient contents. The addition of 2 mL of final compost extract, prepared by adding water to compost in a ratio of 5:1, was added to two sheets of sterilized filter paper in a Petri dish (90 mm diameter) and tested for the germination of pakchoi seeds (*Brassica rapa* L.). An increase in plant root length from 77 to 148 mm, GI from 72 to 162%, and seed germination rate from 82 to 100% was noted compared to the control sample. However, since the decomposition rate of lignocellulosic contents present in SCG is very low, researchers are thus now focusing on advanced rapid lignocellulose humification technology for converting SCG into biofertilizer.^97^

Zhu et al.^58^ used an integrated heat/base (KOH) co-activated persulfate (PS)-based advanced oxidation process in one system to accelerate the humification of SCG (Table S2.5). The treated SCGs (TSCG) were found to give an optimum yield of 192 mg/g of fulvic-like acid (FLA; 19.2%) and 45 mg/g of humic-like acid (HLA; 3.96%) under 4% KOH, 1% PS in 1 h at 100 °C. Through quenching and electron paramagnetic resonance experiments, the presence of •OH and SO4•– radicals in the heat/KOH/PS system has been confirmed and found to be responsible for carboxylation, hydroxylation, and Maillard reactions during humification, resulting in higher amounts of −COOH and −OH groups in the FA as compared to SCG. Furthermore, they prepared slow-release nano fulvic-like acid fertilizer (SNFF) by mixing attapulgite (ATP; 0.75 g) with TSCG (0.75 g), showing a good slow release of FLA/HLA especially at pH 3. When SNFF (1.5 g) was spread evenly in a pot designed to test the impact of SNFF on chickweed growth, it resulted in maximum enhancement in wet weight, plant height, and average root length of the plant in comparison to control SCG (0.75 g added) and ATP (0.75 g added) (Figure S1). Fertilizer addition also resulted in an increase in volume and diversity of the soil microbial population as well as the pH of the soil, which rose from 5 to 6.7, making SNFF a good competitor for acid soil amendment.

Kamh and Hedia^98^ extracted humic-like substances (HLS) from SCG utilizing KOH extractant and subsequently used it to prepare a liquid organic-mineral fertilizer enriched with N and P in addition to K. The optimized parameters, i.e., KOH, SCG-to-extractant ratio, reaction time, and temperature to extract maximum HLS, were noted to be 2.0 N, 1:10, 3 h, and 80°C, respectively. When evaluated, the potential of developed fertilizers [NPK mineral-organic fertilizers (NPK-HLS) having 8.6/5.8/2/0 as K_2_O/P_2_O_5_/N and 5.1 % w/v HLS] with the control one to grow maize showed a considerable increase in plant growth (shoot dry weight from 1.60 to 2.71 g/pot; root dry weight from 0.86 to 1.08 g/plot), NPK availability in soil, and NPK (enhanced N from 25.06 to 72.03 mg/pot; P: 6.18 to 16.59 mg/pot; K: 65.56 to 116.74 mg/pot) uptake by the plant. High-pressure-water and high-temperature (120–200 °C) treatment facilitates the extraction of various water-soluble compounds like organic acids, minerals, and proteins from SCGs.^99^ Utilizing SCGs in different forms supports sustainable agriculture. It has been observed that each conversion method has special benefits, and when used in combination, they can increase the ability of the resulting fertilizers to boost plant productivity and soil health. For example, biochar/hydrochar lacks microbial life and should be inoculated with vermicompost or compost before being used. Similarly, SCG should be precomposed to reduce the phytotoxicity prior to its introduction to the vermicomposting system. SCG can be combined with different carbonaceous materials like cardboard, peanut leaves, rice bran, straw, or eggshells to balance the nutrient content and pH.

## 4 Impact of Spent Coffee Grounds on Plant Growth and Elemental Composition in Plants, and Factors Affecting Plant Growth

### 4.1 Impact on Elemental Composition

SCG can enhance the level of vital nutrients that are necessary for human health in plants because of the presence of some chelating compounds like melanoidins and polyphenols^100,101^ (Table S3). A significant enhancement in elemental concentrations of Fe, Co, V, and Ca has been found for lettuce plants cultivated in soil amended with 1–5% SCG than those grown in soil without SCG^102^ (Table S3). However, N contents, linked to plant growth and productivity, in the plant after SCG amendment have been noted to be diminished, which could be due to its absorption by the microorganisms present in the soils. The average enhancement, compared to control soil, in elements for lettuces grown in 7.5 to 15 and 1–5% doses of SCG were reported to be 32% for Zn, 56% for Mn, 161% for Co, 306% for V, and 191% for Fe. Intake of toxic metals like As and Al from 150 g of lettuce grown in SCG-amended soil rose by 54 and 352% compared to lettuces grown in non-amended soils. It has been estimated that 150 g of lettuce can provide 3.128μg of As and 965μg of Al; these values lie far below or in the range of their provisional tolerable weekly intake limit of 15μg/kg for As^103^ and 200–1500μg/kg for body weight.^104^ Keeflee et al.^85^ reported a non-significant increment in amounts of metals like Cd, Zn, Pb, and Cu for spinach grown in the SCG-based co-compost-amended soil, and their range was noted to be below the permissible limit set by Food Regulations 1985. When 2.5% of SCG was applied in soil amendment, an increment in Ca, Cu, K, P, and N content in cauliflower species, up to 47.1, 57.4, 27.7, 27.1, and 11.7%, respectively, and a decrement in Fe and Na content by 24.2 and 14.9% was observed.^105^ Furthermore, the accumulation of Ca, Na, and Fe for broccoli and P, Na, and Fe for cabbage grown in 2.5% SCG was also confirmed. Zn and Mg were observed to accumulate in cauliflower cases; however, their content decreased in cabbage and broccoli after SCG was applied to soil. Cervera-Mata et al.^91^ observed an increase in Ca and Fe accumulation in lettuce plants from 139 to 160 mg/100 g and from 0.742 to 1.45 mg/100 g, respectively, upon replacement of SCG with SCG-derived hydrochar for treating the soil. Cervera et al.,^106^ in another study, utilized SCG modified with Zn and Fe biochelates and found a significant enhancement in Zn and Fe contents in lettuce plants around 177–416 and 28–30%, respectively. Based on this discussion, it can be inferred that adding SCG to the soil does not raise the nutrient levels above the harmful threshold, making it a potentially beneficial solution to preserve healthy soil.

### 4.2 Factors Impacting Plant Growth

Various factors can impact plant growth, including chemical composition (already discussed in Section 2), pH, salinity, and C/N ratio, which have been discussed below in detail.

#### 4.2.1 Salinity

Compost salinity plays a crucial role in soil fertility, and any values between 0.75 and 1.99 mS/cm support the most favorable conditions for seed germination.^33^ Utilization of amendments possessing higher EC may lead to phytotoxic impacts in plants. Cervera-Mata et al.^94^ reported an increase in EC of SCG from 6.03 to 48 mS/cm after vermicomposting, to 22.55 after composting, 11.95 after pyrolysis to biochar, 11.25 after ethanol treatment, 3.43 after water treatment, and 5.42 for hydrolyzed SCG. Such an increase in EC has been related to enhancements in basic hydroxides after the vermicomposting process. When soil amendments were employed, an increase in soil salinity was observed. The lowest salinity level was observed in the case of soil amended with pyrolyzed SCG (1.34 mS/cm) and raw SCG (1.6 mS/cm). In comparison, the highest levels were observed for soil amended with composted SCG (3.53 mS/cm) and vermicocomposite SCG (4.76 mS/cm). Contrary to the above results, some authors found a decrease in the salinity after vermicomposting. Sanchez-Hernandez and Domínguez^107^ reported a decrease in SCG salinity from 179 ± 25 to 118 ± 7.5 after vermicomposting SCG with an earthworm species, *Eisenia andrei*. Picca et al.^55^ showed EC values ranging from 0.6 to 2.02 dS/m for various peat and compost mixtures with and without biochar, with the biochar-containing combination showing a lower value. Their findings confirm that biochar may help to minimize salinity by retaining nutrients, allowing for higher volumes of compost in growing media.

#### 4.2.2 pH

Soil pH determines the availability of various nutrients to plants. There is an ideal pH range for the optimum presence of different nutrients.^108^ For instance, the soil’s pH ranging between 6 and 7 confirms the optimal availability of P, and 6.5 to 8 is optimal for the presence of macronutrients like K, Mg, Ca, N, and S. At the same time, 5 to 7 is optimal for micronutrients such as B, Cu, Fe, Mn, Ni, and Zn. Outside of these optimal limits, plants have less access to nutrients. Besides molybdenum (Mo), other micronutrient availability declines when soil pH gets closer to 8. This is because cations have a strong binding affinity for soil and are difficult to exchange. Various metals like Mn, Fe, Ni, Cu, and Zn are bound tightly to soil at higher pH levels and thus are readily available at low pH levels.

In addition to this, the tolerance of individual plants and soil organisms varies with the acidic or/alkaline nature of the soil. In general, neutral conditions are highly suitable for plant growth; however, the optimum soil pH range for individual plants varies. Table S4 shows the pH ranges of different SCG samples, their derived biochar and hydrochar, SCG-derived compost, and vermicompost. From the table, it can be concluded that the pH of SCG lies in the range of 4.76–5.52;^61^ SCG-derived biochar,^56,109–111^ mostly basic, lies between 8 and 10; SCG-derived hydrochar,^29,91,110^ acidic by nature, lies between 3 and 4; vermicompost^57,110^ lies in the range 7–8; and for compost,^60,85^ it ranges between 6 and 8, with SCG+ FeSO_4_ compost (80:20) being an exception, whose pH is 1.79.^100^ Numerous efforts have been made to amend the soil using SCG and SCG-derived amendments, and their impact on the soil C/N ratio and pH is detailed in Table S4.

The acidic nature of SCG and SCG-derived hydrochar indicates its suitability as an ameliorant for alkaline soils, whereas biochar may be suitable for acidic soils. The composting and vermicomposting process neutralizes the pH of SCG while simultaneously reducing the tannic, caffeine, and phenolic concentrations and enhancing the availability or maintaining the contents of nitrogen and other minerals.^62^ These results confirm the suitability of composted or vermicompost SCG (since pH varies between neutral and slightly acidic to slightly basic) as soil amenders for all types of plant growth. However, an optimum quantity of selected amendments is necessary, as unnecessary amendments can backfire. An excessive reduction of soil pH might lead to hazardous circumstances, whereas elevation initiates a reaction of nutritional imbalances. Certain microorganisms, such as nitrogen-fixing bacteria and nitrifying bacteria (which convert ammonium to nitrate), perform well above a soil pH of 6. Thus, an optimum pH should be maintained; for example, plants like alfalfa, sugar beet, dry bean, broccoli, leek (acid sensitive), and radish grow well in pH above 6;^61,112^ however, sunflower and viola exhibit optimum growth in pH range 5.5 to 6.5 and 5.0 to 6.0, respectively.^61^ Potatoes also show best growth between 5.0 and 5.5 since rising pH levels increase the prevalence of bacteria that cause common scab infections.^113^

#### 4.2.3 C/N Ratio

The C/N ratio of the soil amendment plays a crucial role in defining compost maturity. A high C/N ratio suggests a lengthy process with carbon and quick nitrogen digestion. However, low C/N indicates the existence of excess nitrogen in SCG, which might be dissipated by ammonia volatilization. Thus, the ideal C/N ratio range is 10 to 35.^114,115^ Ratios higher than 35 induce N net immobilization, whereas low values, i.e., less than 10, signify net N mineralization. Table S4 shows the C/N ratio of different SCG, SCG-derived compost, and vermicompost samples, and various soil samples when amended with SCG-derived amenders. SCG showed a C/N ratio between 25 and 35;^40,61,89^ SCG-derived biochar^56,109–111^ or hydrochar^29,91,110^ exhibited a ratio between 16 and 25; alkaline-extracted SCG showed 216;^29^ compost showed 7 to 10;^51,60,100^ and vermicompost showed 7 to 11.26,^57,110^ and when these amenders were added to the soil, a further decrease in the C/N ratio of the soil mixture was noted.^29,49,57,61,65,91,102,105^ The goal of utilizing vermi technology is to simply bring the amender’s C/N ratio down to match the soil’s C/N ratio, which varies from 10 to 12. Plants can easily absorb soil amendments or organic materials if they possess a C/N ratio close to that of the soil.^116^ It is surprising to note that alkaline-treated SCG and alkaline-treated SCG-derived biochar and SCG and peat mixture showed remarkably high C/N ratios, confirming the non-applicability of these amenders. Picca et al.^55^ found an increase in the C/N ratio on the addition of biochar to the mixture of peat and SCG-derived compost from 13.4–24 to 24.7–27.4, confirming the high stability of biochar. Dimitrijević et al.^76^ reported a decrease in the C/N ratio of SCG-derived compost prepared by composting (91 days) a mixture of medicinal plant waste and SCG (1–10 wt %) utilizing a plant growth-promoting bacteria, such as *Hymenobacter* sp., *Streptomyces* sp., *Bacillus* sp., and *Paenybacillus* sp. A decrement from 34.92 to 14.24% has been reported by them, confirming the compost’s suitability for agriculture.

## 5 Impact of Spent Coffee Grounds and Derived Hydrogels on Physical–Hydraulic Properties of Soil

The water and soil environments are continuously deteriorating, primarily due to variable rainfall intensity/patterns and rising air temperature.^117,118^ One of the main obstacles to sustainable agriculture in dry and semiarid regions and in areas with sandy soils is the unavailability of soil water because of the reduced specific surface area of the substrates, resulting in less water adsorption.^119,120^ Different kinds of soil conditioners produced from industrial, mineral, and organic origin (plant waste, coffee waste, sewage waste, etc.) have so far been employed to treat surface evaporation and water percolation.^120^ These conditioners boost the soil’s capacity to absorb and hold water, improving physical-hydraulic properties, including water storage and infiltration throughout the soil’s depth, and increasing the water’s accessibility to plants.^121,122^

Very few researchers have studied the impact of SCG amounts on various physical properties of soil, such as water holding capacity, soil water content at container capacity (θ_cc_), readily available water capacity (RAWC), gravimetric moisture content, and wilting point. Turek et al.^123^ reported a 31% enhancement in the water retention capacity of sandy loam soil (Brazilian origin) with an increase in SCG doses from 0 to 15%. The mean readily available water capacity (RAWC) and θ_cc_ were noted to increase from 43.2 to 60.3 mm and 0.3963 to 0.4686 m^3^/m^3^, respectively; consequently, an opposite trend was noted in mean drainable porosity after the addition of SCG. The θ_cc_ value of the soil depends upon its structure, bulk density, texture and type, and amount of organic matter contents added/or present.^124^ The increase in the θ_cc_ value in Turek et al.'s study, with enhancement in SCG loading, has been related to changes in soil organic matter contents leading to alteration of θ_cc_. Kasongo et al. observed that the addition of a mixture of coffee husk and coffee pulp to sandy soil can enhance water retention (55–60%) and decrease the percolation regardless of the mixture content.^125^

Cervera-Mata et al.^126^ evaluated the impact of increasing doses of SCG (1, 2, 2.5, 5, 7.5, 10, 12.5, and 15%) on the properties of a smectite-rich clayey soil (collected from the Spanish Mediterranean area). Water retention of soil was noted to be enhanced at field capacity (−33 kPa) and permanent wilting point (−1500 kPa) proportionally to the amounts of added SCG. However, the increase in the wilting point (129%) was reported to be much higher than the field water capacity (38%), leading to a decrease in the volume of plant available water content. Additionally, a nonlinear effect on aggregate size is demonstrated, which has led to an increase in overall porosity and a decrease in soil bulk density. In comparison to the control sample, the inclusion of SCG and simple incubation of the samples raised each aggregate’s individual porosity, noting 430% for the samples containing 5% SCG and 639% for the 15% SCG included sample. Khan et al.^127^ added 1 and 2 t/ha SCG to urban soil at the University of Agriculture in Krakow campus and, after the addition of 3 months, subsequently evaluated the hydrological properties of treated soil. Best hydrological properties were noted with 2 t/Ha addition of SCG and improved the volumetric water content of the soil, when compared to the control one, by 18%; declined the water drop penetration from 4.83 s, in the case of control soil, to 2.33 s; and enhanced the current water storage capacity and water capacity after 4 h and after 24 h by 71, 54, and 54%, respectively. Furthermore, a decrease in soil pH by 7 and 14 % and nitrogen contents by 2 and 9% was noted after the application of 1 and 2 t/ha SCG to the soil. Alghamdi et al.^93^ applied SAWB and SCWB, obtained by pyrolysis of coffee waste at 550°C, in varying proportions of 1 to 5 wt %, to loamy sand soil in a column set up. Application of SAWB and SCWB at 5% declined the cumulative infiltration and cumulative water evaporation by 57–66 and 124–181%, respectively, and enhanced the water retention in the range of 101 to 130% in comparison to the control sample (without any amendments). A decrease in the soil bulk density and hydraulic conductivity after adding biochar was also noted because of the change in pore distribution and aggregate stability of the soil. The lowest hydraulic conductivity was observed for 5% SCWB and SAWB-added soil samples, reaching 0.020 and 0.019 cm min^–1^, respectively. Hardgrove and Livesley,^61^ during the greenhouse trial, reported an increase in the water-holding capacity of sandy and silty soils after the addition of fresh SCG. Similarly, the gravimetric moisture content enhancement was also observed when the SCG dose was varied ≥10% during the soil field trial. Contrary to above results, Cervera-Mata et al.^128^ noted a considerable increase in contact angle and water drop penetration time, i.e., enhance in hydrophobicity on addition of raw SCGs to two different soils (vega and red soil).

Hydrogels possess excellent water-absorbing and retention properties because of the availability of numerous hydrophilic groups and modest crosslinking. SCG-derived hydrogels have been mostly used for the adsorption of heavy metals and dyes from contaminated water and the generation of freshwater.^129–132^ Recently, alkali-treated SCG and coal fly ash (CFA) were utilized to prepare novel and low-cost [SCG-polyacrylic acid/CFA] by making use of the aqueous solution polymerization technique,^133^ with modified coffee grounds (MCG), acrylic acid (AA), and CFA as raw materials, and it was applied to soil to improve its drought resistance. Various parameters were optimized (optimized parameters are alkali treated SCG: 6 wt %; CFA: 2 wt %; acrylic acid: 5 g; KPS: 0.6 wt %; MBA: 0.14 wt %; time: 2 h) to obtain maximum water absorbency capacities of 82 and 1260 g/g in physiological saline and deionized water, respectively. When 0.1 wt % alkali-treated SCG-PAA/CFA hydrogels were employed as soil conditioners, they exhibited water-holding capacities of 3.76, 4.42, and 6.65% in clay soil, loam soil, and sandy soil, respectively. Still, research on utilizing SCG to prepare hydrogels for agricultural applications is in the primary stage. The long-term impact of SCG-derived hydrogels on soil health, i.e., plant growth and soil nutrient supply, should be a part of future research. Some parameters such as wilting point, field capacity, and plant available water contents of different hydrogels and their performance under different soil stress conditions should also be thoroughly evaluated for commercialization in the agriculture field.

## 6 Conclusions

Coffee, one of the most consumed drinks worldwide, generates millions of tons of residues, including SCG solid waste. It contains a huge amount of organic and inorganic compounds; that is why it has been utilized to create lucrative byproducts in various industries. The valorization of SCG is decreasing the amount of landfill waste and creating new green jobs. However, efforts are required from the waste management companies to introduce separate waste collection (equipment, transport, bins and containers network, labor charges, infrastructure for decomposing, etc.), new collection routes, new valorization techniques, etc.^33^ For successful implementation of SCGs as fertilizers, supply chain logistics and economic considerations must be carefully considered. The SCG is generated continuously and abundantly by centralized and decentralized sources like coffee industries and coffee shops and households, respectively. Centralized sources offer efficient collection points, while decentralized sources may pose logistical challenges. Additionally, restaurant- and café-generated SCGs are meant to be used on agricultural land in rural or regional locations. Therefore, we should establish composting units near markets, coffee shops, or agroindustrial areas to reduce transportation costs. Moreover, information and communication technologies need to be utilized to optimize collection schedules and routes, which will save transportation expenses and boost productivity. SCG’s high moisture content is another drawback that raises transportation expenses and necessitates rapid processing or energy-intensive drying to avoid spoiling.

From a technoeconomic perspective, inexpensive SCG is a desirable alternative for upcycling into products with added value, such as hydrogels, compost, biochar, and biopolymers. However, preprocessing procedures, including extraction, grinding, and drying, may increase operating expenses. Cooperation between government agencies and private businesses to finance infrastructure could encourage the use of SCG-based fertilizers.

Spoilage and mold contamination pose persistent operational risks for SCG. Since SCG possesses a high moisture content (about 65%), if it is not adequately dried or composted, then it may be susceptible to mold contamination and microbiological spoiling.^134,135^ The SCG contamination by more specifically *Aspergillus* species could produce well-known mycotoxins such as aflatoxins, ochratoxin A, and gliotoxin and cause aspergillosis. Thus, contamination of SCG by these species could pose a high risk to both human health and the environment.^136,137^ Furthermore, poor management and storage of SCG can lower its overall quality. If stored at 35 °C, then microbial populations may expand exponentially, which subsequently might degrade the SCG and result in the production of short-chain fatty acids like butyric, acetic, and formic acids.^138^ The lipids that are found in SCG can also potentially auto-oxidize to produce these chemicals.^139^

Furthermore, SCG is commonly utilized as fertilizers as such or after treatment to enhance the fertility of the deteriorating soil, caused by deflocculating processes, the inaccessibility of basic cations such as Mg^2+^ and Ca^2+^, and the presence of excess sodium ions.^36^ However, enhancement in soil fertility depended upon the nature of SCG, since fresh SCG causes serious effects on plant growth and is generally applied for controlling weeds. Numerous techniques have been employed by researchers, such as composting, vermicomposting, conversion to biochar or hydrochar, alkali treatment, and extraction of humic-like substances, for the conversion of phytotoxic SCG into environmentally friendly fertilizers (Figure 3).

**Figure 3:**
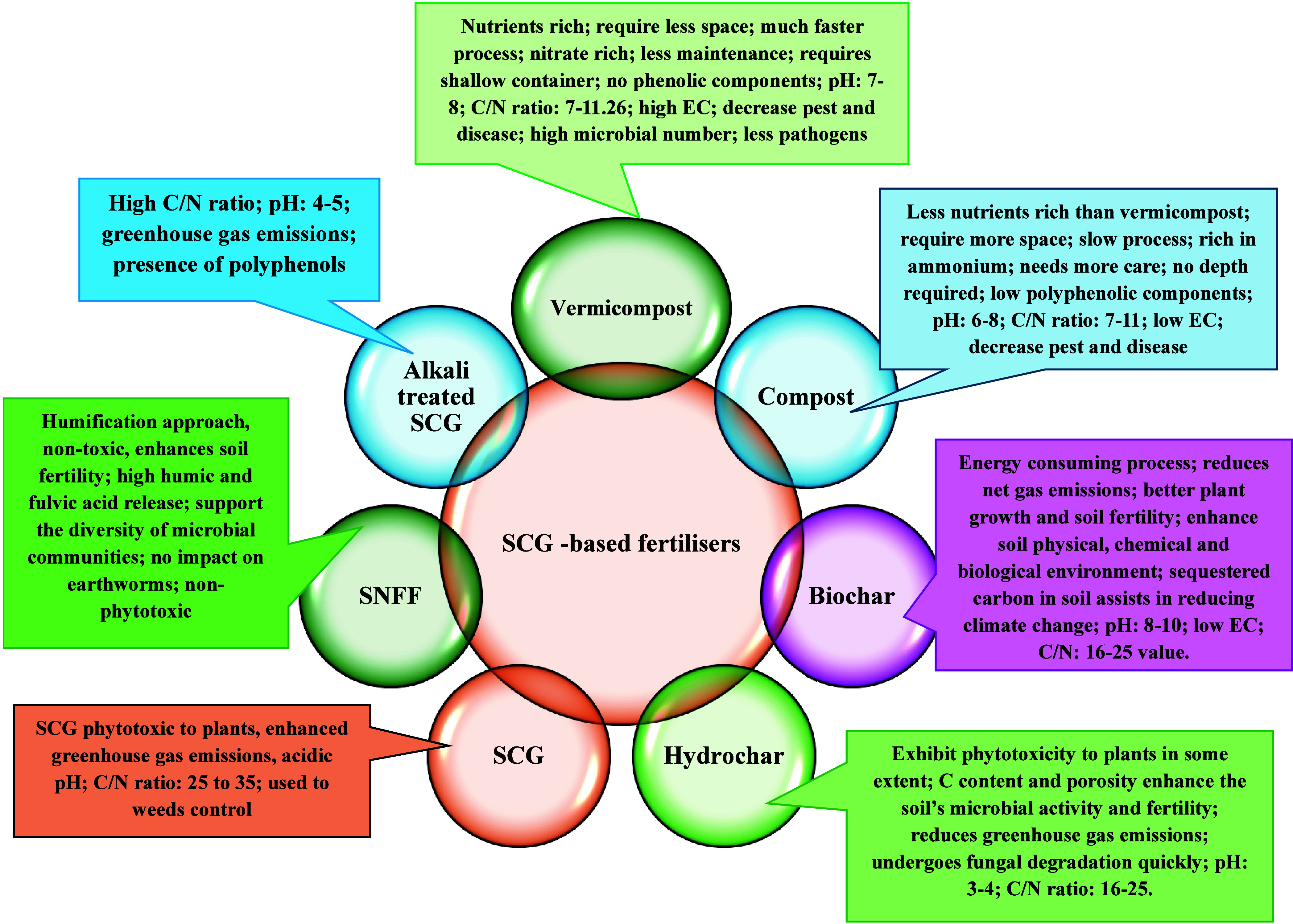
Different SCG-based fertilizers and their properties and application benefits.

Among the different techniques, vermicomposting is an eco-friendly and cleaner approach; however, it still faces several hurdles in its wider application and acceptance. Since vermicompost SCG possesses high EC, it may impact soil quality, particularly in arid or semi-arid regions. Besides collecting the SCG wastes from different sites, waste management companies should train and guide local farmers on how to vermicompost the SCG at the field and what optimized amount of vermicompost should be applied in the target field. Furthermore, vermicomposting is a longer process than composting, and thus, in-depth research is needed to shorten the vermicomposting timing. Conversion of SCG to biochar (carried out by pyrolysis in the presence of limiting oxygen) or hydrochar (carried out in an autoclave in the presence of water) is a cost-effective and quite impressive technique, as both possess low EC.^140,141^ However, they are energy-consuming processes and need proper training about the soil-crop environment factors and amount and frequency of these materials to be used in soil amendments, as biochar is basic. In contrast, its counterpart, hydrochar, is acidic by nature. Future research is still needed on their long-term application and harmful effects, evaluating the life cycle in soil–crop environment, economic analysis, studying the greenhouse gas emissions (if any), and quantifying the persistence of biochar and soil carbon sequestration capability. When used to remediate the soil, SNFF showed some very encouraging outcomes. However, the process is time-consuming and expensive, and the study is still conducted under lab conditions. Although extract from several SCG-derived bioadditives was also used as a bioamender, many studies were focused on the evaluation of their effect on plant seed germination. It ought to be assessed for plant growth in the future. Lastly, it was observed that the alkali-treated SCG amender had a high pH and C/N ratio; thus, it is not generally recommended for soil amendment.

Using SCG after treatment or after hydrogel conversion significantly increased the soil’s water-holding capacity. However, further research is required to determine factors like available water contents and wilting point, after adding bioamendment to soil. Future research should focus on the long-term effects of these hydrogels on soil fertility.

## Supplementary Material



## Notes

(Figure S1) Chickweed plant growth in slow-release nano fulvic-like acid fertilizer (SNFF)-amended soil (figure reprinted); (Table S1) chemical and mineral composition of SCG; (Table S2) impact of amendment of soil with different SCG-derived organic fertilizers on seed germination, seedling, and plant growth; (Table S3) elemental composition of grown plants; (Table S4) comparative view of the pH and C/N ratio of SCG-derived fertilizers and soil amended with these fertilisers; and references (PDF)
