# Supplementary material for: Sustainable Valorization of Spent Coffee Grounds: A Green Chemistry Approach to Soil Amendment and Environmental Monitoring
Source: ACS Sustain Resour Manag. 2025 Sep 16;2(9):1630–42. doi: 10.1021/acssusresmgt.5c00083 (PMC12478863; doi:10.1021/acssusresmgt.5c00083)
Supplement: Supplementary file 1 [file rm5c00083_si_001.pdf]

Supporting Information for

Sustainable Valorization of Spent Coffee Grounds: A Green Chemistry Approach to Soil Amendment and Environmental Monitoring

Ashvinder Kumar[a], Manju K. Thakur[b], Phil Hart[c,d] and Vijay K. Thakur[a]*

*[a]Biorefining and Advanced Materials Research Center, SRUC, Kings Buildings, West Mains Road, Edinburgh, EH9 3JG, UK. *E-mail: Vijay.Thakur@sruc.ac.uk*

[b]Dept of Chemistry, RNT Govt College, Sarkaghat, Distt Mandi, HP, 175024, India

[c]The School of Water, Energy and Environment (SWEE), Cranfield University, MK43 0AL, UK

[d]Renewable and Sustainable Energy Research Centre, Technology Innovation Institute, P.O. Box 9639, Abu Dhabi, United Arab Emirates

**E-mail: Vijay.Thakur@sruc.ac.uk*

Table of Contents	Page no
**Fig. S1** (A) Photographs of pant growth, (B) wet weight, (C) dry weight, (D) plant height, (E) I chlorophyll content, (F) root length, and (G) survival rate of chickweeds with different treatments. (H) Soil pH variation curve during cultivation. (I) Bacterial communities at genus level in soil of blank and SNFF groups after harvest	2
**Table S1.** Comparative view of chemical composition (w/w% dry basis) and mineral composition of some SCGs studies reported by different researchers (mg/kg).	3
**Table S2.** Comparative view of growth, seed germination and seedling of plant, grown in soil amended with different SCG based amendments.	4- 8
**Table S3.** Showing the elemental composition in plants grown in SCG derived fertilisers amended soil.	9
**Table S4.** This shows the comparative view of the pH and C/N ratio of SCG, SCG-derived biochar, compost, vermicompost, hydrochar, and soil amended with these fertilisers.	10
**References**	11-15

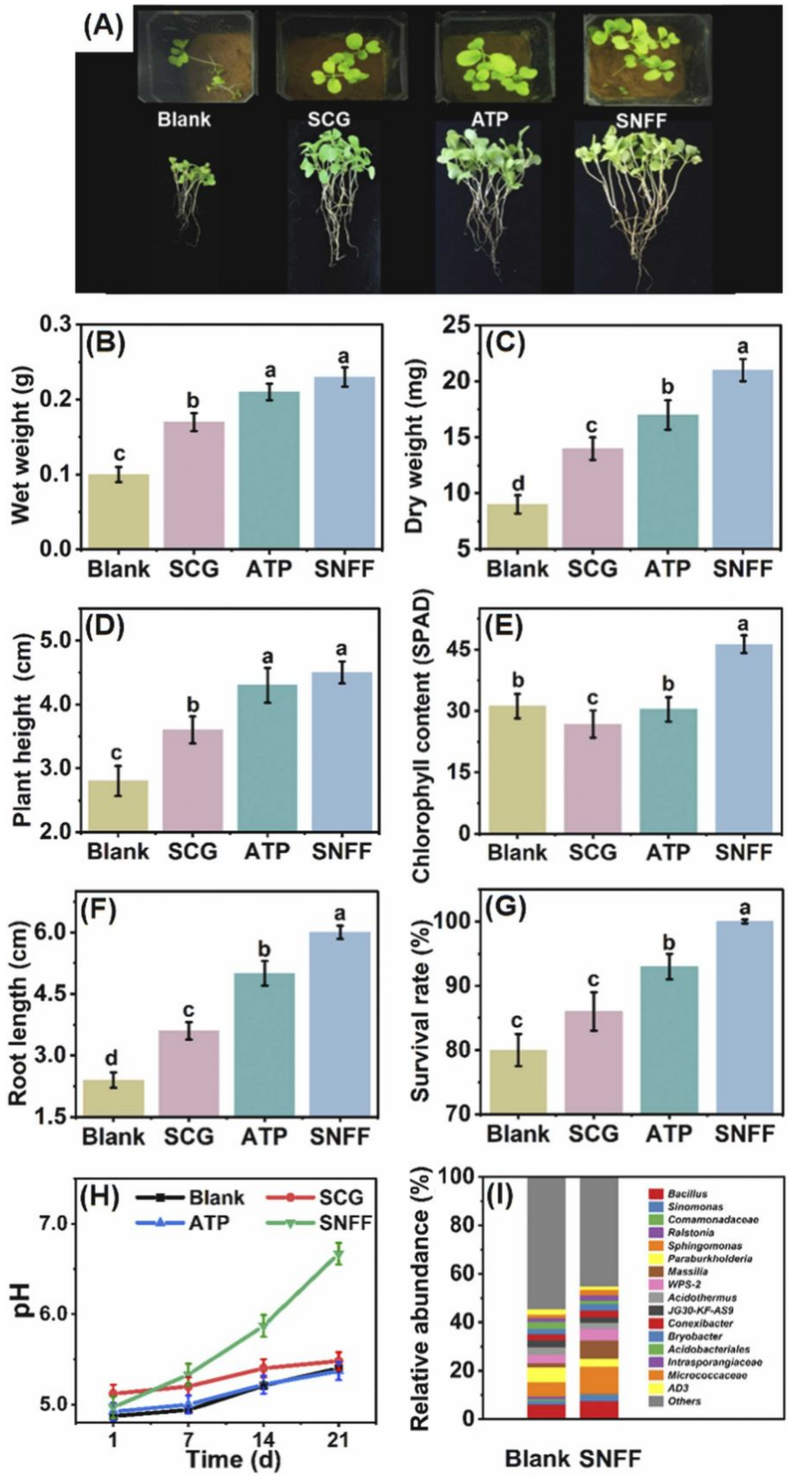

Fig. S1 (A) Photographs of pant growth, (B) wet weight, (C) dry weight, (D) plant height, (E) I chlorophyll content, (F) root length, and (G) survival rate of chickweeds with different treatments. (H) Soil pH variation curve during cultivation. (I) Bacterial communities at genus level in soil of blank and SNFF groups after harvest [1]. Reprinted with permission from Ref. [1] Copyright 2023, Elsevier.

Table S1. Comparative view of chemical composition (w/w% dry basis) and mineral composition of some SCGs studies reported by different researchers (mg/kg).

Chemical/mineral composition	SCGs [2]	SCGs [3]	SCG [4]	SCGs [5]	SCGs [6]	SCGs [7]	SCGs [8]	SCGs [9]	SNFF [1]	SCG [10]	SCG derived biochar [11]	Vermicompost [10d]	Compost of SCGS [12a]	Compost of SCGs [13b]	Compost of SCGs [14c]	Compost [8e]	Compost [8f]
Cellulose	--	13.8	14.8	11.61-13.19	--	8.6	--	--	--	--	--	--	--	--	--	--	--
Hemicellulose	--	--		37.16-41.04	37.06–40.80	36.7	--	--	--	--	--	--	--	--	--	--	--
Lignin	--	33.6	31.02	22.20-25.60	19.84–26.51	--	--	--	--	--	--	--	--	--	--	--	--
Ash	6.2	2.2	1.91	1.20-1.40	--	1.6	--	--	--	--	--	--	--	--	--	--	--
Total extractives	--	--		--	51.43–55.78	--	--	--	--	--	--	--	--	--	--	--	--
Protein	11.5–16.5	13.7	10.7	17.34-17.54	--	13.6	--	--	--	--	--	--	--	--	--	--	--
Lipids	15.3–15.9	--	--	--	--	--	--	--	--	--	--	--	--	--	--	--	--
Total Phenolics	--	----	--	--	0.17–4.54	--	--	--	--	--	--	--	--	--	--	--	--
Dietary fiber	--	-	--	58.27-62.65	--	-	--	--	--	--	--	--	--	--	--	--	--
**pH**	--	--	--	--	--	--	4.97 ± 0.10	6.4	--	5.8	9.4 ± 0.1	7.03	--	6.1 ± 0.2	6.4 ± 0.03	5.70 ± *0.05*	5.63 ± *0.02*
**K**	--	--	--	11,700± 0.01	215-253	3549.0	4820-5300	85.37±0.86	160± 2.6	3072	2700 ± 100	12.34	1.66 %	--	11100	4990 ±20	4860 ±160
**Ca**	--	--	--	1,200±0.00	498-771	777.4	1460-2080	1214.90 ±15.95	--	--	40000 ± 3500	--	--	--	15600	35980 ±3640	32270 ±1100
**Mg**	--	--	--	1,900±0.00	073-178	1293.3	1460-1500	127.06 ±0.56	--	--	1900 ± 100	--	--	--	4600	1760 ±60	1540 ±20
**S**	--	--	--	1,600±0.00	--		830-910	--	--	--	4600 ± 800	--	--	----		840± 40	1260 ±20
**P**	--	--	--	1,800±0.00	--	1475.1	1490-1570	9.20 ±1.34	16.8± 0.54	228	1600 ± 300	7.17	3.36 %	--	2000	1490 ±20	1500 ±20
**Al**	--	--	--	22.30±3.50	--	279.3	--	--	--	--	--	--	--	--	10300	--	--
**Fe**	--	--	--	52.00±0.50	147-326	118.7	--	--	--	8.21	--	0.074	--	--	900	--	--
**Na**	--	--	--	33.70±8.75	329-627		100-160	--	--	--	2144 ± 1.0	--	--	--	216.2	200± 30	25O± 30
**Mn**	--	--	--	28.80±0.70	029-033	40.1		--	--	--	--	0.029		--	117.65	--	--
**Zn**	--	--	--	8.40±0.20	10-12	15.1		--	--	14.87	184.5 ± 1.5	0.034	61.99	133.4	51.00	--	--
**Co**	--	--	--	15.18±0.05	--			--	--	--	--	--		--		--	--
**Cu**	--	--	--	18.66±0.94	39-46	32.3			--	47.63	208.5 ± 5.5	0.025	16.27	48.22	49.45	--	--
**Ni**	--	--	--	1.23±0.59	--	--		--	--	--	216.3 ± 13.7	--	1.05	5.79	12.60	--	--
**C**	--	--	--	--	--	--	490640-510040	--	--	--	--	--	--	1,341 ± 87	437200 ± 3400	466090±8030	477170±6190
**N**	--	--	--	--	--	--	24540-26680	--	53±1.1	--	--	2.5	2.41 %	548 ± 21	27300 ± 700	23620 ±440	24740 ±600

[a]Compost (24 gm) + soil (1.2 kg) [SCG (47%) + biochar (3%) + rice bran (50%), and functional microorganisms [Bacillus sp. (SB-3 and SB-4 +Streptomycessasae (St-3)]; [b] SCG(80%) +poultry manure (20%)+ microbial agent (0.2%); [C] 50% Peat +

50 % compost (SCG, and green waste (1:1 *v/v*); [d][Compost of SCG + cow manure] (1:1) + worms; [e] [SCG and Chicken eggshells mixture in ratio 100:10]; [F]SCG and duck eggshell mixture in ratio of 100:10

Table S2. Comparative view of growth, seed germination and seedling of plant, grown in soil amended with different SCG based amendments.

Aging/Treatment applied	Top dressed/incorporated	Cultivation time	Crops evaluated	Major findings	Ref.
**Table S2.1 SCG**					
SCG of ≤7 months + soil base (2:1 (soil: peat moss) [1:3 SCG to soil)	incorporated	30 Days for radish and 20 days for tomato	radish and tomato growth	• Inhibited plant growth and development<br>• reduced herbivory	15
Vega soil + 2.5 and 10% SCG (w:w)	Mixed	60 days	Little Duende	• Lettuce growth decreased by 233% compared to control one.	10
Red soil+ 2.5 and 10% SCG (w:w)	Mixed	60 days	Little Duende	• Lettuce growth decreased by 201 % compared to control one.	10
SCGs of varying concentration SCG-0 (0g), SCG-5 (5g), SCG9 (9g), and SCG-14 (14 gm) furrowed 2 inch deep and 2 inches away from the plant and then covered using topsoil	Mixed	45 days	Solanum lycopersicum (tomato)	• In comparison to SCG9 and SCG-14, SCG5 exhibited best plant growth, means higher dosage led to overfertilization.<br>• SCG-5 outperformed SCG0 in terms of tomato plant growth, indicating its potential as a fertilizer.	16
SCGs (10 kg/m^2 plow in paddy field)	Mixed	5 months	Weed: Gramineae, wingleaf primrose-willow and horseweed	• Plow-in application of 10 kg /m2 of SCG every 4 month was more effective for germination of weed seed in an upland field converted from a paddy field.<br>• SCG had a greater the inhibitory impact on the growth of Gramineae weeds compared to Asteraceae and Onagraceae.<br>• SCG enhances the levels of soil nutrients, and can be utilised to achieve better crop yields, as well as weed growth inhibition in the subsequent periods.<br>• SCG did not show any the plant growth inhibitory impact longer than 4 months.	17
SCGs (20 kg/m^2 in mulching application)	Mulching application				
SCG (2.5 to 20%) + alluvial sandy soil	Incorporated	77 days	viola, radish, sunflower, broccoli and leek	• SCG significantly reduced plant growth for all horticultural plant<br>• With the increment of SCGs (5 to 20%) amount, a significant decrement in weeds growth noted.<br>• Increasing SCG amount also increased soil water holding capacity.	18
Soil + addition of SCG in varying proportions (17, 33 and 50%)	Mixed	35 days	Brassica rapa	• Soil containing 17% SCG resulted in highest growth of plant (2.1 cm) after 5 weeks followed by control sample (2 cm), 50% SCG (1.3 cm) and 33% SCG containing soil (0.8 cm).<br>• Maximum mass of plant was recorded when grown in control samples (0.090 g) succeeded by 17% (0.083 g), 50% (0.081 g) and 33% (0.035 g) SCG containing soils.	19
Topsoil + 4, 8, 12 and 16% of SCG	Mixed	12 days	Rye (Secale cereale L.)	• Addition of 4 and 8 wt. % of SCG enhanced the number of nitrogen assimilating and spore-forming bacteria.<br>• The plant stem length, weight, and number of leaves grown in control samples were reported to be higher than those grown in a SCG containing medium.	20
SCG concentration of 5 and 10 kg/m2 in sandy loam soil	Top dressing after the seed germination	3 years	Wheat-soybean double cropping	• At SCG concentration of 5 kg/m2 or higher, the soil total nitrogen and carbon contents were noted to enhance significantly after the first application (approx. after 20 months.<br>• Apart from the first wheat cropping, SCG showed no discernible impact on crop yield.<br>• During cropping, a 10 kg/m2 SCG application lowered the biomass of weeds by 50% or more, with the exception of the second-year wheat crop.	21

SCG of varying composition 0.5, 1 and 2.5 gm	Top dressing	35 days	Organic Red Radish seeds	• The SCG, when used for top dressing, give the best result in comparison to mixing techniques.<br>• Among different top-dressing amounts, the plant grown in soil dressed with 1 gm of SCG showed best fresh weight (27.54 gm); while grown in 2.5 gm SCG dressed soil exhibited highest dry weight (5.09 gm) and leaf measures (7.88).	22
SCG of varying concentration (5, 10. 25 and 50%) mixing in general media	Mixing	35 days	(Ravanello Cherry Belle)		
Soil + SCG at rates that satisfied recommended (1.0; $SCG_{1.0}$), double the recommended (2.0; $SCG_{2.0}$), half the recommended (0.5; $SCG_{0.5}$) the and nitrogen requirements of Leaf lettuce (15 Mg N/ha) and Japanese hog fennel (10 Mg N/ha)	Added	One month	Leaf lettuce and Japanese hogfennel seedling transported to pots after 4 weeks)	• SCGs beat NPK in every aspect examined for Japanese hogfennel, but had a negative influence on leaf lettuce.<br>• SCGs2.0, SCGs1.0 and SCGs0.5 amended soils, when compared to control treatment lowered the leaf lettuce's fresh shoot weights by 83.32, 60.04 and 75.57 %, respectively.	23
SCG (varying amount 2.5; 5, 10%, 15 and 20%) + vegetable soil	Mixed	39 days	32 days aged Lettuce plants were transferred to pot containing a mixture of SCG and soil	• Leaves biomass was noted to decrease with increase in SCG contents.<br>• Maximum carotenoids (19.53 ± 2.28 mg/100g) and chlorophylls (25.01 ± 2.26 mg/100g) contents in plant was observed at 15 wt.% of SCG concentration.	24
SCG in different proportions (2-8 %)	Mixed	6 weeks	Fragaria vesca	• 4% SCG addition has been found to possess highest average and growth rate for plant.	25
**Table S2.2 Old SCG**					
14 months old SCG + soil base (clay-loam soil mixed with peat moss) [1:3 SCG to soil)	incorporated	30 Days for radish and 20 days for tomato	radish and tomato growth	• promoted growth<br>• no effect on herbivory	15
8 months old SCG [1 cm top dressing]	Top-dressed	30 Days for radish and 20 days for tomato		• promoted growth<br>• Reduce slug herbivory	15
SCG (3 months old) in varying proportions with sand (0:100; 10:90; 20:80 and 30:70)	Mixed	120 days	(Pinus pinea) Seedlings	• Maximum seedling hight was noted in 100% sand (11.4 cm) followed by SCG + sand mixture (20:80;11.1 cm).<br>• The root collar diameter (3.9 mm) was noted to be maximum in 20:80 mixture of SCG and sand.	26
**Table S2.3 Biochar and hydrochar**					
Sandy-Loam topsoil + Raw/Pretreated SCGs primary biochar (0, 10, 20, 50 and 100 t/Ha) prepared through hydrothermal carbonisation at different temperature (217, 223 and 260°C) and soil liquid extraction	Mixed (topsoil)	28 days	Arabidopsis thaliana (Arabidopsis) and germination of Barbarea verna L.	• Alkaline pre-treated SCG derived primary biochar showed outstanding performances when applied at 100 t/ha: resulted in increase in Arabidopsis thaliana dry weight, leaf number, fresh weight and rosette diameter by 976, 118, 2726 and 531%, respectively, in comparison to control.<br>• Petri-dish assay was used to study the toxicology of SCG and SCG derived biochar on the germination of Lactuca sativa L. seeds.<br>• Toxicity assays of SCG based primary chars with Barbarea verna L. revealed a maximum of 35% increment in root length compared to the control.	27
SCGs derived biochar (amount of water added to biochar containing Petri dish is equivalent to 0 to100 t/ha on a volume basis at a depth of 10 cm in soil)	Seeds grown in Petri dish containing biochar solution	24 hrs (incubation)	Lactuca sativa	• GI noted to decrease from 100.0 to 42.2 with an enhancement in biochar rate from 0 to 100 t/ha.	28
SCG (3 months old) in varying proportions with sand (0:100; 10:90; 20:80 and 30:70)	Mixed	120 days	(Pinus pinea) Seedlings	• Maximum seedling hight was noted in 100% sand (11.4 cm) followed by SCG + sand mixture (20:80;11.1 cm).<br>• The root collar diameter (3.9 mm) was noted to be maximum in 20:80 mixture of SCG and sand.	26
loamy sand soil + 0, 1, 2, 3 and 5% of biochar derived from Spent Arabica coffee (SACB) and Columbian coffee (SCCB) wastes (after subjecting pyrolysis process at 550 °C)	Mixed	30 days (green house conditions)	Maize (Zea mays L.	• 5% application of SACB and SCCB enhanced the water retention ability of sandy loam by 101 to 130%.<br>• 5% application of SACB and SCCB amendments caused an increase in root biomass from 0.12 (control: 100% sandy loam) to 2.12 and 2.38 g, plant highest from 15.71 to 30.94 cm and 32.23 cm, shoot biomass from 7.37 g to 9.70 and 9.93 g respectively.	29
1 and 2.5 % of SCG and SCG derived hydrochar with agricultural soil from the Vega of Granada	Mixed	--	30-day-old lettuces	• Both result in inhibitions in plant growth and causes enhancement in the Mg, Fe, Ca, Cu, and Mn contents.	30

SCGs (5, 10, 25 and 50%) with seed starter potting mixture (SCGPF) [applied from the first day]; SCGs on the top-dressing fertilizer (SCGTDF) [applied after 2 or 3 weeks after planting seeds]	SCGPF : Mixing; and SCGTDF : top dressing	35 days	red radish (*Raphanus sativus*) growth	• 0.5 g SCGTDF treatment yielded the highest mean plant length (18.47 cm) and fresh weight (27.54 g).<br>• The highest average root dry weight of 0.55 ± 0.08 g/plant was recorded in the SCGTDF 1.0 g, and the lowest was recorded in the SCGPF 10<br>• Highest average total plant dry weights of 7.738 ± 1.49, was noted for SCGTDF 2.0 g.<br>• The highest root fresh weight (2.65 ± 0.66 g/plant) was noted for SCGTDF 1.0 g and the lowest in case of SCGPF 5, 10, and 25 %<br>• Highest total plant fresh weight (g) was noted for SCGTDF 1.0 g	31
**Table S2.4 SCG derived compost and vermicompost**					
1-3% SCG compost (prepared by adding fungi starter) + soil	Mixing	21 days	Mustard plant	• Mustard plant showed the best germination index value of 200.4% with 3% SCG compost.<br>• Further, compared to commercial compost and compost control, plants grown in 3% SCG compost added soil also showed best growth.	32
1kg/m^2 application of i) Fresh SCG (100: 1 mixture of SCGs and Trichoderma sp.) + triple dose of liquid worm fertiliser (SCGLWF); and ii) composted SCG [SCG and peanut leaves and branches (10:1 w/w): 1% Trichoderma sp.]+ triple dose of liquid worm fertiliser (CSCGLWF)	Top-dressing	42 days [(SCGLWF and CSCGLWF) applied three 10, 20 and 30 days after planting]	B. campestris	• The CSCGLWF resulted in the best B. campestris yield (3,866.7 g/plot), succeeded by the SCGLWF, (3,766.7 g/plot).<br>• The lowest yield of 2,100.0 g/plot was noted when treated with 1 kg/m^2 fresh SCG with no LWF.<br>• Further, B. campestris L. best height of 29.10, and foliage diameter of 47.00 cm was noted with SCGLWF and CSCGLWF, respectively.	33
Universal soil composed of peat (1.2 kg)+ Eco-friendly composts (24 gm) prepared using varying amount of SCGs (and their extracts), rice bran, biochar, and functional microorganisms (plant growth-promoting bacteria i.e., *Bacillus cereus* SB-3 and *Bacillus toyonensis* SB-4, and plant pathogen inhibiting actinomycetes i.e., *Streptomyces sasae* St-3)	Mixing	17 days	Pepper	• Noted higher TEAC (36.2%) and total flavonoid content (11% better than control) in pepper leaves grown in compost made up by SCG (1.46 kg): Defatted Rice Bran (0.283 kg): biochar (0.045 kg): microbial agent (0.004 kg): Bacillus sp. (1.7 ml): Streptomyces (1.7 ml).<br>• The compost extract treated radish seeds exhibited best seedling growth even in the presence of the pathogen like *Fusarium oxysporum f. sp. lactucae. T*<br>• Best germination index (297%) and wt. per plant for radish and pepper was noted with compost made up by SCG (25.85kg): rice bran (27.25kg): biochar (1.65 kg): microbial agent (0.11 kg): Bacillus sp. (1.5 lit): Streptomyces (1.5 lit).	12
Peat sand+ 2.5, 5 and 10% (v/v) of SCGs and fertiliser made up varying mass proportion of SCG (8-10)+ ash (0-2); produced by low-temperature combustion of biomass) + magnesium sulphate (0-0.9) + blood meal (0- 1)+ gelatine (1). [K12 (9:0.1:0.9:0:1); k13 (8: 0.3: 0.7: 1: 1); K20 (8: 2: 0: 0: 1)]	Mixed	3 days	wheat, mustard, cucumber and garden cress	• 5% addition of SCG and K12, K13 and K20 fertilizers stimulated the development of the different species' roots.<br>• Strong inhibition of seedling growth was noted for samples containing 10% of the fertilizers.<br>• Garden cress was most sensitive, and cucumber was the least sensitive against fertilisers.	34
Topsoil + varying concentration of fresh or composted SCG [fresh SCG: 2.5-20%, v/v and composted SCG: 5-30%, v/v]	Mixed	39 days	Lactuca sativa L.	• The plant growth was noted to be significantly enhanced at 10% and 30% of fresh SCG and composted SCG concentration, respectively, when compared to control.<br>• Use of SCG at concentrations above 10% has been linked to stress on plant growth. This behaviour may be due to increment of phytotoxic compounds (such as caffeine), causing reduction in uptake of macronutrients and thus poor plant growth.<br>• The acceptable concentration of fresh SCG was found to be 2.5 and 5%, v/v; whereas composted SCG should be utilised at concentrations ≥10%, v/v).	35
Peat + Varying concentration (100, 50, 30 or 15%) of SCG composted mixture with biochar (SCG-BC-PGW: SCG, biochar and pruning green waste in ratio 1:1:1 v/v/v) or without biochar (SCG-PGW; SCG and pruning green waste in ratio 1:1 v/v)	Mixed	Seeds germination : 0–6 days; seedling: 7–49 days; and plant-to-fruit maturity: 36–100 days	Tomato (Solanum lycopersicum)	• The tomato growth was noted to be 4.4 to 15-fold lower for SCG-BC-PGW and SCG -PGW growing media in comparison to 100% peat (control).<br>• Seed germination index (GI) was found to maximum in case of SCG-BC-PGW (GI: 331.4 ±8.5) and SCG- PGW (GI: 271.4 ± 4.7) growing media.<br>• The tomato plant, grown (36 days) in peat-based substrate and subsequently shifted to SCG-BC-PGW or SCG-PGW substrate, showed an overall of 100% survival rate.<br>• After the 63-days, except for SCG- PGW 100%, all other substrates led to the best growth of plant, registering a maximum of 88.3 and 80.7cm with SCG-BC-PGW 100% and SCG-BC-PGW 50%, respectively.	14

				• SCG- PGW 50% substrate exhibited 100.3% higher productivity than the 100% peat i.e., control sample.<br>• Fresh fruit biomass was noted maximum for SCG-BC-PGW 50% (60.8%) followed by SCG-BC-PGW 30% (47.1%), SCG- PGW 30% (32.24%) and SCG- PGW 15% (30.75%).	
Soil+ 5 wt.% cat manure and SCGs compost (SCG-CM;1:3 w/w mixture)	Mixed	29 days	*Spinacia oleracea*	• Upon addition of SCG-CM compost an increase in stem height (3 to 19 cm), no of leaves (4 to 12), width of leaf (approx. 0.9 to 6 cm), length of leaf (1 to 7.2 cm approx.) when compared to control sample (100% soil) was noted.	36
Compost [SCG + poultry litter]	Top-dressing	2 years	Bermudagrass (Cynodon dactylon)	• When SCGs was used as top dressing it produces no response as a fertiliser.<br>• However, when used along with poultry litter, a considerable improvement in turf quality relative to synthetic and other organic commercial fertilizers was examined.	37
Sandy loam + 0.1, 0.2 and 0.3% of Iron loaded SCG (Fe-SCG)	Mixed	10 days	White radish (Raphanus sativus)	• Different Fe-SCG loaded soil samples led to improved growth (with Fe-SCG fertilizer began to grow on the third day) of white radish in comparison to control one.<br>• The plant height and growth were found to be maximum with 0.2% ratio treatment than others.	38
3Kg of sandy soil + 1kg or 2Kg of fresh and composted (using Bucket method) SCG	Mixed	Germination after 14 days and plant growth after 12th week	Strawberry growth and fruit quality	• The soils amended with 2Kg composted SCG exhibited superior impacts on strawberry growth (germination %: 95.56 after 14 days), yield and fruit quality (fresh fruit weight:7.907; fruit size: 8.083 cm; 92.18 flower per plants).<br>• Soil mixed with 2kg of composted SCG resulted in highest plant growth (approx. 22 cm) after 12th week of the experiment than the other treatments.	39
Soil + 5, 10 and 15 wt. % of bio mixture of SCG and egg shells [10:2 (w/w) ratio]	Mixed	3 months	Okra	• Maximum yield of Okra was found in the soil amended with 5 and 10 wt. % of bio mixture (noted 161 and 167 g/plant/pot, respectively).<br>• Okra stem diameter, leave numbers and height was noted to be better for 5 wt. % bio mixture treated soil than other bio mixtures amended soil.	40
Soil 10.5 kg) + varied amount (53, 105, 158 and 210 g) of SCG derived compost (made up by mixture of SCG, rice bran, rice husks in a ratio of 2:1:1)	Mixed	Seedling for 30-36 days	Tobacco plants	• Application of SCG compost in the lowest amount resulted in the maximum growth of plant compared to higher SCGs derived compost.<br>• However, a significant decrement in plant growth was noted when compared to control treatment.	41
Sandy clay loam (3 kg) + SCGs derived fertilisers	Applied with irrigation water	6 weeks after sowing	Maize	• NPK-HLS fertilisers of different P rates, 125 (NPK-HLS1) and 250 mg (NPK-HLS2) P/pot without basic fertilizers were used.<br>• Both fertilisers result in significantly enhancement in NPK uptake and availability in soil and shoot dry matter, compared to the untreated sample.	42
Pre-fermented SCG (6 months) used as 9 t/ha	Mixed	86 days	Maize	• Fresh root weight, dry root weight, root volume, plant height and stem diameter were observed to increase from 122.79 to 203.41 g, 23.60 to 35.67 g, 457.00 to 653.04 ml, 1.84 to 2.21 m, and 2.39 to 2.99, respectively.<br>• An increase in metabolic quotient(1.5), microbial biomass (219.01) and organic matter was also reported.	43
i) Fresh SCG (1 kg/m^2; obtained after mixing with Trichoderma sp. In 100:1 w/w/ ratio) + liquid worm fertilisers (LWF: 0.3 mL/m^2) and ii) compost SCG (composting 10:1 ratio w/w mixture of SCG and peanuts branches and leaves with Trichoderma sp. For 45 days) + LWF (0.3 mL/m^2)	Top dressing (applied three times for every 10 days after planting)	42 days	B. campestris L. plants	• Composted SCG + LWF combination showed best plot yield (3,866.7 g/plot), followed by fresh SCG + LWF (3,766.7 g/plot).<br>• However, nonsignificant increment in height, number of leaves and foliage diameter of plant was observed with fresh SCG + LWF combination than composted SCG + LWF.	33
Adding a varying amount (1 -3 wt. %) of SCG compost to soil (compost prepared by inoculating a mixture of SCG, chicken manure and cow dung, with *Penicillium sp. And Aspergillus sp.* starters	Addition	21 days	Compost was applied during the vegetative phase of the Mustard Plants (Brassica juncea L)	• The compost with starter fungi exhibited a higher GI value (200.4%) than with commercial starter (164.84%).<br>• On addition of 3% SCG compost plant growth and nutrients uptake were noted to be maximum.	32
Vega soil + 7.5 % of SCG derived compost (composting of 28.72 kg of SCG with 8.5 kg of dolomite [$CaMg(CO_3)_2$]	Mixed	40 days	lettuces (Lactuca sativa var. Longifolia)	• Highest increase in plant wet mass was found with vermicompost (20 gm) followed by biochar (10.5 g), compost (3.61 ± 0.41 g), fresh SCG (2.22 ± 0.41 g), ethanol-SCG, water-SCG and hydrolyzed SCG.	44

)/ vermicompost (SCG treatment for 8 months with *Eisenia foetida*) / biochar (pyrolysis of SCG at 400°C)/ ethanol treated, water treated and hydrolyzed SCG				• Vermicompost and biochar showed maximum growth for plant but diminished the amount of Zn, Cu and Fe in lettuce because of microbial /thermal degradation of natural chelating components (polyphenols).	
SCGs vermicompost (0, 15, 20, 25 gm; formed by adding two kg of SCG and one kg fermented cow dung, and twenty worms) + 5 kg soil (1: 1 v/v mixture of ultisol soil and sand)	Mixed	3 months	Capsicum frutescens L.	• Soil containing 15 g of vermicompost showed best seed germination ability.<br>• Soil containing 25 g of vermicompost was found to give best growth to Capsicum frutescens L., possessing a maximum ht. of 30 cm, number of leave of 27 and number of branches of 17; while with 0% vermicompost was found to be 20 cm, 18 and 9, respectively.	[45]
**Table S2.5. Extract of torrefied SCG and SCG based compost, and nanofertilisers**					
Maize straw+ SCG and Maize straw+ SCG + yeast effluent compost	Extract	72 h	Sinapis alba and Triticum L	• Both composts were found to be toxic, but Maize straw+ CG+ yeast extract compost was found to be most toxic to S. alba and Triticum L, then Maize straw + SCG compost.	[46]
Extract of SCG and SCG torrefied at 250, 300, 350, 400, 450 and 500°C	Extract	48 h (incubator)	Lepidium sativum L. seeds	• The extract prepared by using SCG torrefied at 350°C, when used for germination of plant showed germination index higher than 50%, confirm that SCG350 can be applied for soil amendment.<br>• Non-torrefied and SCG torrefied at 250 ^{o}C were found to be most toxic.<br>• Further, SCGs treated at higher temperatures has been found to inhibit seed germination because of breaks down of various naturally occurring substances.	[47]
SCG compost (25 weeks co-composting of SCG, poultry manure and olive mill wastewater sludge mixture and inoculating with the *Trametes versicolor)*	Extract used	48 hr	Germination of seed of L (*L. sativa*) and H (*H. vulgare*)	• GI was noted to be 100 and 120% for lettuce and barley after 25 weeks of composting.<br>• Inoculation process enhanced the humification process by 1.25-fold than the control one, i.e., uninoculated compost.<br>• Inoculation also resulted in 72% reduction in polyphenols contents as compared to control one, while prior to inoculation only 36% reduction was noted.	[48]
SCG compost [Composting mixed medicinal plant waste with mixture (2% v/w) of *Paenybacillus sp*., *Streptomyces sp*., *Hymenobacter sp*., and *Bacillus sp.,* and SCG (5% w/w)]	Extract used		Germination of Fagopyrum esculentum seeds	• Showed 50% increment in GI value in comparison to the control sample (only medicinal plant waste-based compost).<br>• The developed compost samples exhibited stability under all i.e., biological, chemical and physical parameters.	[49]
2ml of compost (prepared by addition of SCG and Cow dung to green waste and subsequently inoculating with *Trichoderma* spp. (60%, v/v) and *Phanerochaete chrysosporium* (40%, v/v))	Extract used	48 hr	Brassica rapa L. seed germination	• A considerable increase in plant GI from 72 to 162 %, root length from 77 to 148 mm, and seed germination rate from 82 to 100% was noted when compared to control sample	[50]
Acid soil (pH: 5; 300 gm) + SNFF (1.25 gm)	Spread evenly to the soil surface	21 days	Chickweeds	• When compared to control one, SNFF enhanced (approximately) the chickweeds' root length (increased from 1.5 cm to 6.0 cm), plant height (from 2.7 to 4.5 cm), wet weight (0.1 to 0.22 gm), dry weight (from 7 to 22 mg).	[1]

Table S3. Showing the elemental composition in plants grown in SCG derived fertilisers amended soil.

Plant/SCG/compost amount			Fe (mg/kg)	Co (µg/g)	V (ng/g)	Mn (mg/kg)	Cu (mg/kg)	Zn (mg/kg)	*As (ng/g)	*Pb (µg/g)	*Cd (µg/g)	*Al (µg/g)	N (g/kg)	K (g/kg)	P (g/kg)	Mg (g/kg)	Ca (g/kg)	Na (g/kg)
Baby Lettuce after cultivation for 40 days [51] [1-5% SCG in vega soil]		0	2.0 ± 0.98	4.5 ± 1.5	.7 ± 6.0	1.4 ± 0.36	0.40 ± 0.01	0.67 ± 0.08	13 ± 5.2	0.0089 ± 0.0047	0.237 ± 0.073	1.4 ± 1.4	1.1 ± 0.13	1.377 ± 0.141	0.094 ± 0.015	0.109 ± 0.023	0.489 ± 0.108	--
		SCG	6.0 ± 2.6	11 ± 4.3	35 ± 18	1.4 ± 0.42	0.46 ± 0.07	0.92 ± 0.19	20 ± 5.1	0.013 ± 0.005	0.133 ± 0.097	7.2 ± 3.8	0.72 ± 0.20	1.283 ± 0.201	0.097 ± 0.015	0.111 ± 0.026	0.512 ± 0.112	--
Spinach plant after cultivation for 29 days in SCG-cat manure (3:1 wt.%) -amended soil (5 % w/w wet basis) [36]		0	--	--	--	--	0.87 ± 0.04	2.30 ± 0.14	--	0.63 ± 0.09	0.23 ± 0.02	--	--	--	--	--	--	--
		SCG	--	--	--	--	1.11 ± 0.24	0.83 ± 0.05	--	0.67 ± 0.10	0.25 ± 0.06	--	--	--	--	--	--	--
Brassica species after cultivation for 45 days (2.5 wt.% SCG+ 97.5 wt. % peat) [52]	Cauliflower	0	38.12	--	--	--	47.15	55.03	--	--	--	--	17.12	22.79	5.10	4.37	4.44	8.11
	Cauliflower	2.5 wt. % SCG	34.63	---	--	--	53.10	65.66	--	--	--	--	17.64	23.55	5.43	4.98	4.98	7.05
	Broccoli	0	35.28	---	--	--	71.81	99.52	--	--	--	--	20.31	23.24	5.75	6.42	7.53	10.16
	Broccoli	2.5 wt. % SCG	61.16	---	--	--	68.08	75.59	--	--	--	--	21.98	29.35	6.35	6.19	8.98	13.54
	Cabbage	0	60.45	---	--	--	74.92	182.15	--	--	--	--	20.25	31.23	7.39	8.28	6.52	11.30
	Cabbage	2.5 wt. % SCG	69.35	---	--	--	84.05	103.12	--	--	--	--	22.52	32.84	7.64	5.04	7.85	15.93
Lettuce plant grown in SCG-Fe/Zn bio-chelate - soil mixture for 40 days (2g bio-chelate/kg of soil) [53]		2 g SCG	7.95 ± 0.76					1.06 ± 0.15										
		2 g SCG-Fe	8.57 ± 0.89	ND	ND	ND	ND	ND	ND	ND	ND	ND	ND	ND	ND	ND	ND	ND
		2 g SCG-Mn	ND	ND	ND	ND	ND	4.85 ± 0.30	ND	ND	ND	ND	ND	ND	ND	ND	ND	ND
Lettuce plant growth in SCG based hydrochar-*soil mixture after 40 days* [30]		0	07.37 ± 01.56	---	--	02.87 ± 0.2	06.66 ± 0.10	---	--	--	---	--	5.000	6.060± 0.100	--	0.1812 ± 0.0058	1.280 ± 0.070	0.0482 ± 0.0051
		1% hydrochar	19.95 ± 04.21	---	--	03.02 ± 0.25	32.50 ± 05.12	---	--	--	---	--	4.500	5.390 ± 0.060	--	0.2623 ± 0.0042	1.590 ± 0.110	0.1267 ± 0.0301
		2.5 % hydrochar	17.29 ± 05.56	---	--	04.20 ± 0.13	31.66 ± 04.92	---	--	--	---	--	4.700	5.520 ± 0.090	--	0.2575 ± 0.003	1.680 ± 0.010	0.2376 ± 0.0552

Table S4. This shows the comparative view of the pH and C/N ratio of SCG, SCG-derived biochar, compost, vermicompost, hydrochar, and soil amended with these fertilisers.

Sample	pH	Moisture content (% w/w dry basis)	C%	N%	C/N	Ref
Six different SCGs samples	4.76 - 5.52	11.5 (approx.)	47.85 to 49.10	2.03 to 2.19	22.15 -24.06	18
Exhausted coffee grounds	1.69 mmol/g acidic group		57.16-59.77	1.18-1.32	52.83-56.51	6
7.5–15% SCG in soil	Increase from 7.0 ± 0.3 to 7.9 ± 0.0 after application		--	0.07	--	51
SCG + Cat manure compost	8.20 ± 0.18	2.59 ± 0.12	--	7.0	--	36
Pre fermented SCG	6	--	39-45.5	3.0 – 3.5	13	43
10% SCG + 90% soil	6.68 ± 0.03	--	2.08 ± 0.10	0.22 ± 0.01	9.45 ± 0.15	26
20% SCG + sandy soil	5.9 approx.	Approx. 16%	--	--	-	18
SCGs derived biochar	9.9	4.16	74.25	3.1	23.95	54
SCGs derived biochar	8.4	2.53	69.8	4.253	16	55
SCG derived biochar	9.42	5.2	87.38	4.28	20.42	56
SCGs derived hydrochar	3.9	3.46	62.6	2.470	25	55
SCGs derived vermicompost	7.8	10.38	40.6	5.77	7	55
SCGs derived biochar	9.4 ± 0.1	1.3 ± 0.2 % (m/m)	31.9 ± 0.8	1.69 ± 0.0	18.87	11
Alkaline extracted SCGs	4.72 ± 0.09	4.50 ± 0.16	47.68 ± 0.23	0.22 ± 0.02	216.82 ± 23.81	27
Alkaline extracted SCGs-derived hydrochar based primary char	3.77 ± 0.07	3.75 ± 0.01	58.06 ± 0.30	0.65 ± 0.12	88.71 ± 16.13	27
Hydrochar derived from alkaline SCGs + Soil (100 tn/ha)	7.9-8.1	---	7.9-8.1	0.63-0.66	12.31.12.54	27
SCG (2.5 wt. %) + peat (97.5 wt. %)	4.86 ± 0.01	--	57.51	0.51	111.07	52
SCG (2.5%) + Vega soil	7.9 (vega soil pH: 8.2)	--	2.96	0.138	21	10
SCG (2.5%) + Red soil	7.1 (red soil pH: 7.9)	--	2.31	0.148	16	10
SCG (2.5%) + Vega soil after 60 days of lettuce cultivation	8.4	--	2.56 ± 0.51	0.247 ± 0.078	11 ± 1	10
SCG (2.5%) + red soil after 60 days of lettuce cultivation	7.4	--	2.20 ± 0.18	0.155 ± 0.006	14 ± 1	10
SCGs+ $FeSO_4$ compost (80:20)	1.78	--	391.4 mg/g	41.5 mg/g	9.4	57
Hydrochar derived from SCG	3.95	3.20	58.73	2.16	27	30
Hydrochar derived from SCG (1%) + vega soil	8.25 ± 0.01	--	2.44 ± 0.01	0.22 ± 0.01	11 ± 1	30
Hydrochar derived from SCG (2.5%) + vega soil	8.12 ± 0.01	--	3.47 ± 0.05	0.25 ± 0.01	14 ± 1	30
Compost (Mass proportions of SCG: ash: magnesium sulphate: blood meal: gelatine=8:0.3: 0.7: 1:1)	6.39	--	45.15	4.18	10.80	34
Vermicompost [Compost of 40 kg of SCG + cow manure (1:1) + 4 kg of worms]	7.03		28.15	2.5	11.26	58
4 % Vermicompost [Compost of 40 kg of SCG + cow manure (1:1) + 4 kg of worms] + 5 kg of planting medium (30% soil + 30% rice husk + 20% cocopeat + 15% biochar + 5% lime)	6.4	--	0.66	0.24	2.75	58

Compost [25 weeks co-composting of SCG, poultry manure and olive mill wastewater sludge mixture and then inoculating with the *Trametes versicolor*]	--	--	--	-	14 ± 2	48

References

(1) Zhu, Y.; Zhang, K.; Hu, Q.; Liu, W.; Qiao, Y.; Cai, D.; Zhu, P.; Wang, D.; Xu, H.; Shu, S. Accelerated Spent Coffee Grounds Humification by Heat/Base Co-Activated Persulfate and Products' Fertilization Evaluation. *Environ. Technol. Innov.* **2023**, *32*, 103393.

(2) Daniel, T. Use of Spent Coffee Ground (SCG) as Ingredient in Bread Formulation. *Addis Ababa Univ.* **2018**.

(3) Caetano, N. S.; Silva, V. F. M.; Melo, A. C.; Martins, A. A.; Mata, T. M. Spent Coffee Grounds for Biodiesel Production and Other Applications. *Clean Technol. Environ. Policy* **2014**, *16* (7), 1423–1430. https://doi.org/10.1007/s10098-014-0773-0.

(4) Caetano, N. S.; Caldeira, D.; Martins, A. A.; Mata, T. M. Valorisation of Spent Coffee Grounds: Production of Biodiesel via Enzymatic Catalysis with Ethanol and a Co-Solvent. *Waste Biomass Valorization* **2017**, *8* (6), 1981–1994. https://doi.org/10.1007/s12649-016-9790-z.

(5) Ballesteros, L. F.; Teixeira, J. A.; Mussatto, S. I. Chemical, Functional, and Structural Properties of Spent Coffee Grounds and Coffee Silverskin. *Food Bioprocess Technol.* **2014**, *7* (12), 3493–3503. https://doi.org/10.1007/s11947-014-1349-z.

(6) Pujol, D.; Liu, C.; Gominho, J.; Olivella, M.; Fiol, N.; Villaescusa, I.; Pereira, H. The Chemical Composition of Exhausted Coffee Waste. *Ind. Crops Prod.* **2013**, *50*, 423–429.

(7) Mussatto, S. I.; Carneiro, L. M.; Silva, J. P.; Roberto, I. C.; Teixeira, J. A. A Study on Chemical Constituents and Sugars Extraction from Spent Coffee Grounds. *Carbohydr. Polym.* **2011**, *83* (2), 368–374.

(8) Tombarkiewicz, B.; Antonkiewicz, J.; Lis, M. W.; Pawlak, K.; Trela, M.; Witkowicz, R.; Gorczyca, O. Chemical Properties of the Coffee Grounds and Poultry Eggshells Mixture in Terms of Soil Improver. *Sci. Rep.* **2022**, *12* (1), 2592.

(9) Cruz, S.; Marques Dos Santos Cordovil, C. S. Espresso Coffee Residues as a Nitrogen Amendment for Small-Scale Vegetable Production: Coffee Residues for Vegetable Production. *J. Sci. Food Agric.* **2015**, *95* (15), 3059–3066. https://doi.org/10.1002/jsfa.7325.

(10) Cervera-Mata, A.; Pastoriza, S.; Rufián-Henares, J. Á.; Párraga, J.; Martín-García, J. M.; Delgado, G. Impact of Spent Coffee Grounds as Organic Amendment on Soil Fertility and Lettuce Growth in Two Mediterranean Agricultural Soils. *Arch. Agron. Soil Sci.* **2018**, *64* (6), 790–804. https://doi.org/10.1080/03650340.2017.1387651.

(11) Carnier, R.; Coscione, A. R.; Delaqua, D.; Abreu, C. A. de. Coffee Industry Waste-Derived Biochar: Characterization and Agricultural Use Evaluation According to Brazilian Legislation. *Bragantia* **2021**, *80*, e5721.

(12) Santhanarajan, A.-E.; Han, Y.-H.; Koh, S.-C. The Efficacy of Functional Composts Manufactured Using Spent Coffee Ground, Rice Bran, Biochar, and Functional Microorganisms. *Appl. Sci.* **2021**, *11* (16), 7703.

(13) Emmanuel, S. A.; Yoo, J.; Kim, E.-J.; Chang, J.-S.; Park, Y.-I.; Koh, S.-C. Development of Functional Composts Using Spent Coffee Grounds, Poultry Manure and Biochar through Microbial Bioaugmentation. *J. Environ. Sci. Health Part B* **2017**, *52* (11), 802–811. https://doi.org/10.1080/03601234.2017.1356165.
(14) Picca, G.; Goñi-Urtiaga, A.; Gomez-Ruano, C.; Plaza, C.; Panettieri, M. Suitability of Co-Composted Biochar with Spent Coffee Grounds Substrate for Tomato (Solanum Lycopersicum) Fruiting Stage. *Horticulturae* **2023**, *9* (1), 89.
(15) Horgan, F. G.; Floyd, D.; Mundaca, E. A.; Crisol-Martínez, E. Spent Coffee Grounds Applied as a Top-Dressing or Incorporated into the Soil Can Improve Plant Growth While Reducing Slug Herbivory. *Agriculture* **2023**, *13* (2), 257.
(16) Baranda, R. A.; Rodolfo, C. L. M. The Effect of Spent Coffee Grounds to the Growth of Solanum Lycopersicum (Tomato). **2021**.
(17) Hirooka, Y.; Kurashige, S.; Yamane, K.; Kakiuchi, M.; Miyagawa, T.; Iwai, K.; Iijima, M. Effects of Different Application Methods of Spent Coffee Grounds on Weed Growth. *Weed Technol.* **2022**, *36* (5), 692–699.
(18) Hardgrove, S. J.; Livesley, S. J. Applying Spent Coffee Grounds Directly to Urban Agriculture Soils Greatly Reduces Plant Growth. *Urban For. Urban Green.* **2016**, *18*, 1–8.
(19) Iljas, B.; Bell, D.; Nguyen, E.; Tran, S. The Effects of Used Spent Coffee Grounds on the Growth of Brassica Rapa. **2019**.
(20) Ragauskaitė, D.; Šlinkšienė, R. Influence of Urea on Organic Bulk Fertilizer of Spent Coffee Grounds and Green Algae Chlorella Sp. Biomass. *Sustainability* **2022**, *14* (3), 1261.
(21) Hirooka, Y.; Kurashige, S.; Yamane, K.; Watanabe, Y.; Kakiuchi, M.; Ishikawa, D.; Miyagawa, T.; Iwai, K.; Iijima, M. Effectiveness of Direct Application of Top Dressing with Spent Coffee Grounds for Soil Improvement and Weed Control in Wheat-Soybean Double Cropping System. *Plant Prod. Sci.* **2022**, *25* (2), 148–156. https://doi.org/10.1080/1343943X.2021.2007142.
(22) Mesmar, A.; Albedwawi, S.; Alsalami, A.; Alshemeili, A.; Al Raish, S. Effect of Spent Ground Coffee, Vermicompost and Chemical Fertilizers on Organic Red Radish (Ravanello Cherry Belle) Plant and Soil Quality. **2023**.
(23) Jeon, Y.-J.; Kang, Y.-G.; Eun, J.-A.; Oh, T.-K. Yield, Functional Properties and Nutritional Compositions of Leafy Vegetables with Dehydrated Food Waste and Spent Coffee Grounds. *Appl. Biol. Chem.* **2024**, *67* (1), 22. https://doi.org/10.1186/s13765-024-00863-0.
(24) Cruz, R.; Baptista, P.; Cunha, S.; Pereira, J. A.; Casal, S. Carotenoids of Lettuce (Lactuca Sativa L.) Grown on Soil Enriched with Spent Coffee Grounds. *Molecules* **2012**, *17* (2), 1535–1547.
(25) Gray, B. The Effect of Spent Coffee Grounds on the Growth Rate and Dry Mass of Fragaria Vesca Plant. **2018**.
(26) Caliskan, S.; Ozok, N.; Makineci, E. Utilization of Spent Coffee Grounds as Media for Stone Pine (Pinus Pinea) Seedlings. *J. Soil Sci. Plant Nutr.* **2020**, *20* (4), 2014–2024. https://doi.org/10.1007/s42729-020-00271-5.
(27) Massaya, J.; Mills-Lamptey, B.; Chuck, C. J. Soil Amendments and Biostimulants from the Hydrothermal Processing of Spent Coffee Grounds. *Waste Biomass Valorization* **2022**, *13* (6), 2889–2904. https://doi.org/10.1007/s12649-022-01697-x.
(28) Souza, L. Z. M. de; Pinto, B. C.; Alves, A. B.; Ribeiro, A. V. de O.; Feliciano, D. C. T.; Silva, L. H. da; Dias, T. T. M.; Yılmaz, M.; Oliveira, M. A. de; Bezerra, A.

C. da S. Ecotoxicological Effects of Biochar Obtained from Spent Coffee Grounds. *Mater. Res.* **2022**, *25* (Suppl 2), e20220013.
(29) Alghamdi, A. G.; Alomran, A.; Ibrahim, H. M.; Alkhasha, A.; Majrashi, M. A. Spent Coffee Waste-Derived Biochar Improves Physical Properties, Water Retention, and Maize (Zea Mays L.) Growth in Sandy Soil. *Sci. Rep.* **2024**, *14* (1), 19753.
(30) Cervera-Mata, A.; Lara, L.; Fernández-Arteaga, A.; Rufián-Henares, J. Á.; Delgado, G. Washed Hydrochar from Spent Coffee Grounds: A Second Generation of Coffee Residues. Evaluation as Organic Amendment. *Waste Manag.* **2021**, *120*, 322–329.
(31) Mesmar, A. K.; Albedwawi, S. T.; Alsalami, A. K.; Alshemeili, A. R.; Abu-Elsaoud, A. M.; El-Tarabily, K. A.; Al Raish, S. M. The Effect of Recycled Spent Coffee Grounds Fertilizer, Vermicompost, and Chemical Fertilizers on the Growth and Soil Quality of Red Radish (Raphanus Sativus) in the United Arab Emirates: A Sustainability Perspective. *Foods* **2024**, *13* (13), 1997.
(32) Afriliana, A.; Erawantini, F.; Hidayat, E.; Harada, H.; Subagio, A. Assessing the Beneficial Effect of Spent Coffee Ground Compost under Mustard Plants (Brassica Juncea L). *Asian J. Agric. Hortic. Res.* **2023**, *10* (4), 310–321.
(33) Le, T.-L.; Huynh, N.; Green, I. D. Effect of Spent Coffee Grounds and Liquid Worm Fertilizer on the Growth and Yield of Brassica Campestris L. *Acta Fytotech. Zootech. ISSN 1336-9245* **2023**, *26* (4).
(34) Ciesielczuk, T.; Rosik-Dulewska, C.; Poluszyńska, J.; Miłek, D.; Szewczyk, A.; Sławińska, I. Acute Toxicity of Experimental Fertilizers Made of Spent Coffee Grounds. *Waste Biomass Valorization* **2018**, *9* (11), 2157–2164. https://doi.org/10.1007/s12649-017-9980-3.
(35) Gomes, T.; Pereira, J. A.; Ramalhosa, E.; Casal, S.; Baptista, P. Effect of Fresh and Composted Spent Coffee Grounds on Lettuce Growth, Photosynthetic Pigments and Mineral Composition. In *VII Congreso Ibérico de Agroingeniería y Ciencias Horticolas*; SECH e SEAgIng, 2014; pp 1–5.
(36) Keeflee, S. N. K. M. N.; Zain, W. N. A. W. M.; Nor, M. N. M.; Yong, S. K. Growth and Metal Uptake of Spinach with Application of Co-Compost of Cat Manure and Spent Coffee Ground. *Heliyon* **2020**, *6* (9).
(37) Flores, G.; Wherley, B.; McInnes, K.; Feagley, S.; Hejl, R. Evaluation of Spent Coffee Grounds as a Nutrient Source for Turfgrass Systems. *J. Plant Nutr.* **2024**, 1–16. https://doi.org/10.1080/01904167.2024.2380488.
(38) Humayro, A.; Harada, H.; Naito, K.; Hashimoto, A. The Effective Adsorption of Phosphate and Nitrate Using Spent Coffee Ground Loaded Iron and the Effect for Plant Growth. In *6th International Conference of Food, Agriculture, and Natural Resource (IC-FANRES 2021)*; Atlantis Press, 2022; pp 409–417.
(39) Nababi, J.; Murongo, M.; Obeng Apori, S.; Balde, I.; Ssemakula, J. Potential of Coffee Grounds as a Sandy Soil Amendment and Its Effect on Growth and Fruit Quality of Strawberry. **2023**.
(40) Nguyen, N. K.; Nguyen, B. V.; Do, S. H.; Lam, L. T. Effect of Biomixture Containing Spent Coffee Ground and Milled Egg-Shells on the Yield of Okra (Abelmoschus Esculentus Moench) and Soil Fertility under Greenhouse Conditions. *Advence Sci. Eng. Inf. Technol.* **2016**, *4* (6), 495–501.
(41) Bonaventura, A.; Kusumawati, A. Effect of Coffee Grounds as Compost on the Growth of Vorstenlanden Tobacco. *J. Glob. Sustain. Agric.* **2022**, *2* (2), 44–49.

(42) Kamh, M.; Hedia, R. M. NPK-Liquid Fertilizer Based on Humic-like Substances Extracted from Spent Coffee Grounds: Extraction, Preparation and Application to Maize. *Alex. Sci. Exch. J.* **2018**, *39* (April-June), 260–267.
(43) Simões, G.; Demétrio, G. B.; de Paula, G. F.; Ladeira, D. C.; Matsumoto, L. S. Influence of Spent Coffee Grounds on Soil Microbiological Attributes and Maize Crop. *Res. Soc. Dev.* **2020**, *9* (8), e818986400–e818986400.
(44) Cervera-Mata, A.; Navarro-Alarcón, M.; Rufián-Henares, J. Á.; Pastoriza, S.; Montilla-Gómez, J.; Delgado, G. Phytotoxicity and Chelating Capacity of Spent Coffee Grounds: Two Contrasting Faces in Its Use as Soil Organic Amendment. *Sci. Total Environ.* **2020**, *717*, 137247.
(45) Darwis, D.; Lisdiana, H. Application of Vermicompost from Spent Coffee Ground on the Growth of Capsicum Frutescens L. In *IOP Conference Series: Earth and Environmental Science*; IOP Publishing, 2023; Vol. 1228, p 012030.
(46) Kopeć, M.; Baran, A.; Mierzwa-Hersztek, M.; Gondek, K.; Chmiel, M. J. Effect of the Addition of Biochar and Coffee Grounds on the Biological Properties and Ecotoxicity of Composts. *Waste Biomass Valorization* **2018**, *9* (8), 1389–1398. https://doi.org/10.1007/s12649-017-9916-y.
(47) Jeníček, L.; Tunklová, B.; Malaťák, J.; Neškudla, M.; Velebil, J. Use of Spent Coffee Ground as an Alternative Fuel and Possible Soil Amendment. *Materials* **2022**, *15* (19), 6722.
(48) Hachicha, R.; Rekik, O.; Hachicha, S.; Ferchichi, M.; Woodward, S.; Moncef, N.; Cegarra, J.; Mechichi, T. Co-Composting of Spent Coffee Ground with Olive Mill Wastewater Sludge and Poultry Manure and Effect of Trametes Versicolor Inoculation on the Compost Maturity. *Chemosphere* **2012**, *88* (6), 677–682.
(49) Dimitrijević, S.; Milić, M.; Buntić, A.; Dimitrijević-Branković, S.; Filipović, V.; Popović, V.; Salamon, I. Spent Coffee Grounds, Plant Growth Promoting Bacteria, and Medicinal Plant Waste: The Biofertilizing Effect of High-Value Compost. *Sustainability* **2024**, *16* (4), 1632.
(50) Zhang, L.; Sun, X. Using Cow Dung and Spent Coffee Grounds to Enhance the Two-Stage Co-Composting of Green Waste. *Bioresour. Technol.* **2017**, *245*, 152–161.
(51) Cervera-Mata, A.; Navarro-Alarcón, M.; Delgado, G.; Pastoriza, S.; Montilla-Gómez, J.; Llopis, J.; Sánchez-González, C.; Rufián-Henares, J. Á. Spent Coffee Grounds Improve the Nutritional Value in Elements of Lettuce (Lactuca Sativa L.) and Are an Ecological Alternative to Inorganic Fertilizers. *Food Chem.* **2019**, *282*, 1–8.
(52) Chrysargyris, A.; Antoniou, O.; Xylia, P.; Petropoulos, S.; Tzortzakis, N. The Use of Spent Coffee Grounds in Growing Media for the Production of Brassica Seedlings in Nurseries. *Environ. Sci. Pollut. Res.* **2021**, *28* (19), 24279–24290. https://doi.org/10.1007/s11356-020-07944-9.
(53) Cervera-Mata, A.; Fernández-Arteaga, A.; Navarro-Alarcón, M.; Hinojosa, D.; Pastoriza, S.; Delgado, G.; Rufián-Henares, J. Á. Spent Coffee Grounds as a Source of Smart Biochelates to Increase Fe and Zn Levels in Lettuces. *J. Clean. Prod.* **2021**, *328*, 129548.
(54) Tangmankongworakoon, N. An Approach to Produce Biochar from Coffee Residue for Fuel and Soil Amendment Purpose. *Int. J. Recycl. Org. Waste Agric.* **2019**, *8* (S1), 37–44. https://doi.org/10.1007/s40093-019-0267-5.
(55) Cervera-Mata, A.; Delgado, G.; Fernández-Arteaga, A.; Fornasier, F.; Mondini, C. Spent Coffee Grounds By-Products and Their Influence on Soil C–N Dynamics. *J. Environ. Manage.* **2022**, *302*, 114075.

(56) Stylianou, M.; Christou, A.; Dalias, P.; Polycarpou, P.; Michael, C.; Agapiou, A.; Papanastasiou, P.; Fatta-Kassinos, D. Physicochemical and Structural Characterization of Biochar Derived from the Pyrolysis of Biosolids, Cattle Manure and Spent Coffee Grounds. *J. Energy Inst.* **2020**, *93* (5), 2063–2073.
(57) Morikawa, C. K.; Saigusa, M. Recycling Coffee and Tea Wastes to Increase Plant Available Fe in Alkaline Soils. *Plant Soil* **2008**, *304* (1–2), 249–255. https://doi.org/10.1007/s11104-008-9544-1.
(58) Pratiwi, Y.; Saefurahman, G. Vermicompost from Spent Coffee Grounds as a Nutrient-Rich Organic Fertilizer. In *IOP Conference Series: Earth and Environmental Science*; IOP Publishing, 2024; Vol. 1354, p 012011.
